# Distribution-Aware Estimation of A-/B-Basis Characteristic Values for Natural-Fiber Composite Laminates from Single-Source Tensile Data

**DOI:** 10.3390/polym18212713

**Published:** 2026-11-02

**Authors:** Saurabh Tiwari, Jongwon Lee, Mohammad Faseeulla Khan, Nokeun Park

**Affiliations:** 1 School of Materials Science and Engineering, Yeungnam University, Gyeongsan 38541, Republic of Korea; 2 Department of Mechanical Engineering, College of Engineering, King Faisal University, Al-Ahsa 31982, Saudi Arabia; 3 Institute of Materials Technology, Yeungnam University, Gyeongsan 38541, Republic of Korea

**Keywords:** natural fiber composites, A-basis, B-basis, parametric bootstrap, tolerance limits, weibull distribution

## Abstract

Natural-fiber composites exhibit large property scatter, and multi-batch qualification data are rarely available. Using 590 tensile specimens from ten flax, jute, and carbon-fiber laminate groups in a single experimental program, we applied established one-sided lower tolerance limits as approximations of A-basis (99% coverage, 95% confidence) and B-basis (90%, 95%) values for strength, modulus, and failure strain, and compared normal-theory, bootstrap, family-specific pivotal, and selection-robust routes. A Monte Carlo study (eight scenarios, 5000 replicates) showed that the pivotal limit reached a mean A-basis confidence of 0.949 when the family was known but only 0.896 (minimum 0.805) when the family was selected by AIC; an envelope over all three families reached at least 0.944 at the cost of lower limits. An empirical bootstrap cannot exceed a confidence of 1–0.99^*n*^ (0.311 at *n* = 37). Two jute-twill records with a zero video-strain reading at peak load were excluded from the strain analysis by a rule based solely on the measurement record, raising the A-basis failure strain by 45%. Retention rankings partly changed when the sample size was equalized. In the five groups that contain a second file-name series, tensile strength differed significantly between the series (Welch *p* ≤ 0.008), so the pooled limits for these groups assume identically distributed specimens. The values are screening approximations and not design allowables.

## 1. Introduction

Natural-fiber-reinforced polymer composites (NFCs) have attracted sustained interest as lower-density and potentially lower-environmental-impact alternatives to conventional synthetic-fiber composites in transportation, infrastructure, marine, consumer, and semistructural applications. Flax and jute are particularly prominent because they combine low density with useful specific stiffness and strength, and their renewable origin offers additional sustainability benefits to their use. However, the same biological origin that makes these reinforcements attractive also introduces heterogeneity in the fiber geometry, microfibrillar angle, cellulose content, defect population, moisture response, and fiber–matrix interaction. These sources of variability propagate from the fiber scale to yarns, fabrics, and laminates; therefore, the average tensile properties alone are insufficient for reliability-sensitive material selection [1,2,3,4].

The distinction between the average performance and statistically defensible lower-tail performance is central to the composite design. The Composite Materials Handbook (CMH-17) formalizes the A- and B-basis values as one-sided lower-tolerance bounds. The B-basis value is exceeded by at least 90% of the material population with 95% confidence, whereas the A-basis value is exceeded by at least 99% of the population with 95% confidence [5]. These values are more conservative than the sample mean or empirical low percentile because they explicitly account for the finite-sample uncertainty. Under the normal distribution assumption, the lower tolerance limit can be expressed using the sample mean, sample standard deviation, and one-sided tolerance factor obtained from the noncentral t-distribution developed by Owen [6]. CMH-17 also provides lognormal and Weibull basis-value procedures, an Anderson–Darling goodness-of-fit test with an observed significance level, a maximum-normed-residual outlier test, nonparametric procedures, and checks for between-batch variability [5].

For mature aerospace composite systems, formal characteristic value development is embedded in qualification programs involving multiple batches, processing histories, environments, and statistical checks for batch-to-batch variability. Such datasets are rarely available in the NFC literature. Most NFC studies report mean values, standard deviations, coefficients of variation, Weibull parameters, and low empirical percentiles. Virk et al. demonstrated substantial statistical variability in jute fibers and showed that the failure strain can be more consistent than the strength because it is less sensitive to uncertainty in the fiber cross-sectional area [3]. Li and Wang further showed that the statistical nature of natural-fiber strength can materially affect composite strength and failure predictions, and that Weibull-form assumptions can influence the predicted response [4]. These studies establish the need for probabilistic treatment but do not evaluate how reliable basis-value approximations are when only a single-source dataset is available.

A companion study analyzed the same 590-specimen open dataset using the coefficients of variation, empirical fifth percentiles, bootstrap confidence intervals, and AIC-based distribution screening [7]. The present work uses the same specimen-level data and candidate distribution families but addresses a different research question, focusing on the estimation of the first and tenth population percentiles with a specified confidence level rather than the descriptive analysis of the empirical fifth percentile. The statistical methods used in this study are well-established. Normal and log-transformed limits follow Owen [6]; Weibull limits from pivotal quantities of the smallest-extreme-value location–scale family go back to Thoman, Bain, and Antle [8] and are treated in standard texts [9,10] and by Monte Carlo pivotal simulation in [11]. We therefore do not claim a new estimator. The contribution is an application and evaluation of these methods on open NFC data, built around one research question: how much of the nominal 95% confidence of a tolerance limit survives when the distribution family has to be selected from the same small single-source sample, and what does that imply for comparing laminate architectures?

Accordingly, the objectives are to (i) compute A-/B-basis approximations for the strength, modulus, and failure strain of ten laminate groups with several established routes, including a CMH-17-style screening (Anderson–Darling, maximum normed residual, order-statistic bound); (ii) quantify, by Monte Carlo simulation with stored outputs, the achieved confidence of each route under a known family and under AIC family selection, and report conservatism separately from confidence; (iii) show how much the limits move across the plausible families and after the removal of flagged specimens; (iv) apply an eligibility rule for strain records that is based solely on the measurement record and independent of the tolerance-limit results; and (v) show how retention rankings depend on the sample size. The results are screening approximations and do not replace multi-batch qualification.

## 2. Materials and Dataset

### 2.1. Data Source and Material Groups

The analysis used the open tensile test dataset compiled by Torres et al. and distributed through Mendeley Data (DOI: 10.17632/v25pzywt5c.1) [2,12]. The dataset contained 590 specimen-level tensile records covering ten laminate groups and was also used in the companion lower-tail characterization study [7]. The ten groups and specimen counts were Carbon-0 (*n* = 46), Flax-0 (*n* = 40), Flax-90 (*n* = 37), Flax-CP (*n* = 68), Flax-Short (*n* = 41), Flax-Twill (*n* = 54), Flax-VE-0 (*n* = 45), Jute-Plain (*n* = 86), Jute-Satin (*n* = 87), and Jute-Twill (*n* = 86), yielding a total of 590 observations in this study. Carbon-0 is a unidirectional 0° carbon/epoxy reference material. Flax-0 and Flax-90 were unidirectional flax/epoxy laminates tested parallel and transverse to the fiber direction, respectively. Flax-CP is a cross-ply flax/epoxy system, Flax-Short contains randomly oriented short flax fibers, Flax-Twill is a 2 × 2 twill flax/epoxy architecture, and Flax-VE-0 is a unidirectional flax/vinyl ester system. The three jute groups were plain, satin, and twill weave jute/epoxy laminates. Carbon-0 is retained as a synthetic fiber reference rather than a directly interchangeable design candidate.

### 2.2. Measurement Provenance and Eligibility Rule

According to the data article, the specimens were rectangular coupons cut from vacuum-infused laminates and tested on an electromechanical frame following ASTM D3039, with longitudinal and transverse strains measured using an optical (video) extensometer; the crosshead extension recorded in the raw files was not used for material properties, and the Bluehill modulus in the file headers was not used because the modulus was recalculated from the raw stress–strain data over a prescribed initial strain interval [2]. The data article does not state whether the specimens of a material came from one panel or from several panels. Our processed table (Supplementary Table S2) contains the file identifier, strength (stress at peak load), recalculated modulus, failure strain, and video-strain value at peak load for every record.

The failure strain in this study was the axial strain at the peak load, not at the final rupture, and was obtained from the video measurements. In the processed table, each of the 590 records had a video strain value at the peak load; therefore, the extension-based fallback described in the processing workflow was never triggered, and no strain value was mixed between the video and crosshead sources. The crosshead-based strain at the peak was approximately an order of magnitude larger (mean 23% versus 1.6%) because it likely included machine compliance and grip effects, and it was not comparable to the other method. Exactly two of the 590 records, Jute-Twill_Specimen_RawData_38 (2) and Jute-Twill_Specimen_RawData_6 (2), exhibited zero video axial strain at the maximum-stress sample, resulting in processed failure strains of −0.068% and −0.061%, respectively. Direct inspection of the original instrument CSV files confirmed that, for Specimen 38 (2), the video strain decreased from 1.355% at 83.900 s to zero at the maximum stress of 67.03610 MPa (84.012 s), whereas, for Specimen 6 (2), it decreased from 1.415% at 84.200 s to zero at the maximum stress of 69.81103 MPa (84.300 s). The negative processed values are consistent with the subtraction of a small processing offset from these zero readings (median offset approximately −0.04% across the remaining records). These abrupt discontinuities suggest a measurement or synchronization anomaly rather than physically meaningful zero failure strains. Accordingly, an eligibility rule based solely on the measurement record and independent of the tolerance-limit results required positive video strain at peak load, excluding only these two records from the primary Jute-Twill failure-strain analysis (*n* = 84). Their tensile-strength and recalculated modulus values were retained because the load measurement was independent of the video-strain signal and the corresponding moduli (5.78 and 5.85 GPa) remained within the observed Jute-Twill modulus range (5.51–8.00 GPa; Supplementary Table S2). For sensitivity analysis, a separate reconstructed case replaced the two affected strains with 0.9499 times their respective maximum video-strain values, using the median ratio determined from the 84 eligible records. This reconstructed case was evaluated separately and was not included in the primary dataset.

### 2.3. Properties Analyzed and Specimen-Series Structure

Three tensile properties were analyzed: tensile modulus, tensile strength, and axial failure strain. Poisson’s ratio and toughness were excluded. Strain is reported in percent to avoid the loss of significant figures that occur when strain is given to four decimal places. No panel or batch identifier is available. However, 173 of the 590 file names carry a duplicated-file suffix “(2)”, and these occur only in five groups (Flax-CP, Flax-Twill, Jute-Plain, Jute-Satin, and Jute-Twill). We used this suffix as a proxy for a second series of tests and compared the two series within each group (Section 4.8, Supplementary Table S7). The proxy is not documented in the source and we do not interpret it as a batch. We therefore describe the data as single-source, meaning one published experimental program, and treat the independence of coupons as an assumption that cannot be verified from the data.

## 3. Statistical Methodology

### 3.1. Definitions and Normal-Theory Limits

For the material property *X*, a one-sided lower tolerance limit *L*(*p*, *γ*) is a statistic such that, with confidence *γ*, at least a proportion p of the population exceeds *L*. Equivalently, *L* must not exceed the population (1 − *p*) quantile with probability γ over repeated datasets. The A-basis uses *p* = 0.99 (target: the population first percentile), and the B-basis uses *p* = 0.90 (target: the population tenth percentile), both with γ = 0.95 [5]. Here, *p* denotes population coverage and 1 − *p* denotes the lower-tail probability. Because the source is a single experimental program, the results are called characteristic value approximations rather than design allowables. When *X* is normal, the limit is

(1) $$L=\bar{x}-\mathrm{ks}$$

where $\bar{x}$ and *s* are the sample mean and sample standard deviation (divisor *n* − 1) and k is the one-sided tolerance factor, evaluated from Owen’s noncentral-t formulation [6] using

(2) *δ* = *z_p_*√*n*, *k* = *t*_(_*_n_*_−1,_*_δ_*_)_(*γ*)/√*n*

where *z_p_* is the standard-normal quantile, and *t*_(_*_n_*_−1,_*_δ_*_)_(*γ*) is the *γ* quantile of a noncentral t distribution with *n*−1 degrees of freedom and noncentrality *δ*. The tolerance factor *k* is used only for the normal and log-normal constructions; the Weibull shape is denoted as *β*. The factors were checked against tabulated CMH-17 values (*k_B_* = 2.355 for *n* = 10; *k_B_* = 1.697 for *n* = 40; *k_A_* = 3.981 for *n* = 10).

### 3.2. Bootstrap Routes and Their Structural Limit

Three bootstrap constructions were evaluated in this study. Route 2 (parametric bootstrap) fits the AIC-selected family, draws B synthetic samples of size n from the fit, takes the empirical (1 − *p*) quantile (linear interpolation, Hyndman–Fan type 7) within each synthetic sample, and uses the fifth percentile of these B quantiles as the 95% lower bound; the family is not refitted inside the bootstrap. Route 2b (refitted parametric bootstrap) draws the same synthetic samples but refits the selected family by maximum likelihood to each sample and evaluates the analytic (1 − *p*) quantile, which removes the extreme-order-statistic noise of Route 2. Family selection is performed once on the observed data and is not repeated inside the bootstrap; in the simulation in Section 3.5, it is repeated in every synthetic dataset. Route 3 (empirical bootstrap) resamples the observed values with replacement [13] and uses the fifth percentile of the resampled empirical (1 − *p*) quantiles.

Route 3 has a structural limit that can be stated without simulation. Its lower endpoint cannot fall below the sample minimum, and, for independent observations from a continuous population, the probability that the sample minimum lies below the population (1 − *p*) quantile is 1 − *p^n^*. The confidence that any bound not below the sample minimum can attain is therefore at most 1 − *p^n^*: 0.311 at *n* = 37 and 0.583 at *n* = 87 for the A-basis. A nonparametric 99%/95% lower bound requires *n* ≥ 299 (0.99*^n^* ≤ 0.05), and, for the B-basis, the sample minimum is a valid 90%/95% bound from *n* = 29. More resamples cannot remove this limit; it separates a structural information shortage from Monte Carlo instability. As a CMH-17-style nonparametric reference, we also report the distribution-free order-statistic bound *X_(r)_*, with *r* the largest rank for which *P*[Bin(*n*, 1 − *p*) ≥ *r*] ≥ 0.95; this is the rank-based construction and not the Hanson–Koopmans extrapolation [5,14,15]. Bootstrap sizes were B = 4000 for the single-dataset results and B = 2000 (Routes 2 and 3) and 1000 (Route 2b) per replicate in the simulation; convergence is checked in Section 4.6.

### 3.3. Family-Specific Pivotal Limits (Route 4)

Route 4 applies the exact or pivotal tolerance limit of the selected family. For a normal-selected property, it equals Route 1. For a lognormal-selected property, the analysis is performed on *Y* = ln *X*, and the Owen factor of Equation (2) is applied to the sample mean and sample standard deviation of *Y*:

(3) *L* = exp(*ȳ* − *k*
*s_y_* )

For a Weibull-selected property with scale *η* and shape *β*, *Y* = ln *X* follows a smallest-extreme-value (SEV) location–scale model,

(4) *Y* = *μ* + *σ*
*W*, *μ* = ln *η*, *σ* = 1/*β*,

with *W* standard SEV. The population (1 − *p*) quantile on the log scale is

(5) *y*_1−_*_p_* = *μ* + *σ*
*w_p_*, *w_p_* = ln[ −ln *p* ]

For *p* = 0.99, this is the first percentile. With maximum-likelihood estimates

($\hat{\mu }$, $\hat{\sigma }$) = (ln $\hat{\eta }$, 1/$\hat{\beta }$), the lower limit is

(6) $$L=\exp[\hat{\mu }-c(n,\;p,\;\gamma )\;\hat{\sigma }],$$

where *c* is the γ quantile of the pivotal quantity

(7) $$C=(\hat{\mu }-\mu -\sigma \;w_{p})/\hat{\sigma }$$

The distribution of *C* does not depend on (*μ*, *σ*), because ($\hat{\mu }$ − *μ*)/*σ* and $\hat{\sigma }$/*σ* are pivotal for a location–scale family, and *C* = [($\hat{\mu }$ − *μ*)/*σ* − *w_p_*]/($\hat{\sigma }$/*σ*). It can therefore be simulated with the standardized Weibull (*η* = 1, *β* = 1, i.e., *μ* = 0, *σ* = 1), for which *C* reduces to ($\hat{\mu }$− *w_p_*)/$\hat{\sigma }$. We simulated M = 100 000 samples of size n from this standardized population, refitted each by maximum likelihood, and took the γ quantile of *C* as *c*. Then, *P*(*L* ≤ *y*_1−_*_p_*) = *P*(*C* ≤ *c*) = *γ* for any two-parameter Weibull population, which is the property that defines the limit [8,9,10,11]. The calibration factors for every (*n*, *p*) used are stored in Supplementary Table S4; repeating the calibration with five independent random streams gave a standard deviation of *c* below 0.01 for *n* = 37 to 87.

### 3.4. Model Screening and Selection-Robust Envelopes

The normal, lognormal, and two-parameter Weibull families [16] were fitted using the maximum likelihood method, with the location parameter of the lognormal and Weibull fixed at zero; lognormal and Weibull cannot be fitted to non-positive data. AIC = 2*k* − 2 ln *L* with *k* = 2 for each family was computed with the maximum-likelihood scale (divisor *n*); the exact normal and lognormal tolerance factors used the sample standard deviation (divisor *n* − 1). Families within ΔAIC ≤ 2 of the minima are called near-tied. The AIC compares the overall likelihood and does not show that the selected family describes the first or tenth percentile. We therefore also report (a) the CMH-17 Anderson–Darling observed significance level (OSL) for each family [5,17], (b) the iterative maximum-normed-residual (MNR) outlier test at α = 0.05 [5], and (c) two selection-robust limits: Route 5s, the minimum Route-4 limit over all families within ΔAIC ≤ 2, and Route 5a, the minimum Route-4 limit over all feasible families. Probability plots that resolve the lower tail are presented in Section 4.5. The fitted parameters and corresponding model-selection statistics for the candidate distributions are listed in Supplementary Table S3.

### 3.5. Monte Carlo Validation Design

Eight scenarios were defined from the fitted real data parameters: four Weibull, two lognormal, and two normal populations with the sample size of the corresponding dataset. Each scenario used 5000 replicates. Two experiments were conducted using the same synthetic datasets. In the known-family experiment, Route 4 used the generating family. In the AIC experiment, the family was re-selected by AIC in every synthetic dataset, and Routes 2, 2b, 4, 5s, and 5a used the selected family; Routes 1 and 3 did not depend on the family. The achieved confidence is the fraction of replicates with *L* ≤ *q*1−*p*, which is the analytic population quantile. For each route, we reported the achieved confidence with its Monte Carlo standard error, the minimum over scenarios, the mean absolute deviation from 0.95, the number of scenarios with confidence ≥ 0.94, and the median of *L*/*q*1−*p* as a measure of conservatism. Confidence above 0.95 is conservative, and confidence below 0.95 violates the nominal requirement; therefore, the two are assessed separately, and the mean over scenarios is not used as a validation criterion. Every replicate has its own seed sequence (SeedSequence [20261, scenario, replicate]), split into independent streams for the synthetic data and bootstrap; calibration factors use separate streams (Section 3.3). The numerical results presented in Section 4.6 were obtained from a consistent set of Monte Carlo simulation outputs, with the corresponding confidence and convergence summaries provided in Supplementary Table S5. For 5000 independent replicates, the Monte Carlo standard error of the estimated confidence of 0.95 was approximately 0.0031.

### 3.6. Retention and Reproducibility

The characteristic value retention is 100 × (limit/sample mean). Because the tolerance factor depends on *n*, retention was also recomputed at a common *n* = 40 from the fitted parameters of the selected family, which separates the effect of the sample size from that of the scatter. All statistical analyses were performed using Python 3.13 with NumPy, SciPy and pandas. The Supplementary Materials provide detailed characteristic value estimates, specimen-level processed data, fitted distribution parameters, calibration factors, simulation and convergence summaries, outlier analyses, subsampling results, and series comparisons (Tables S1–S7). To support computational reproducibility, an accompanying Code Archive containing Python analysis scripts, unit tests, stored Monte Carlo simulation outputs, and raw-record verification files was provided. Together with the processed specimen-level data and numerical summaries in Supplementary Tables S1–S7, these materials document the computational workflow underlying the statistical estimates, validation results, and sensitivity analyses reported in this study.

## 4. Results

### 4.1. Descriptive Statistics and Distribution Screening

The descriptive statistics and distribution-screening results for the 30 property–group combinations are summarized in Table 1. The analysis considers tensile strength, modulus, and failure strain across ten laminate groups, with the Jute-Twill failure-strain dataset restricted to 84 eligible observations. The results provide an initial assessment of property variability, preferred distribution families, and the uncertainty associated with model selection.

**Figure 1:**
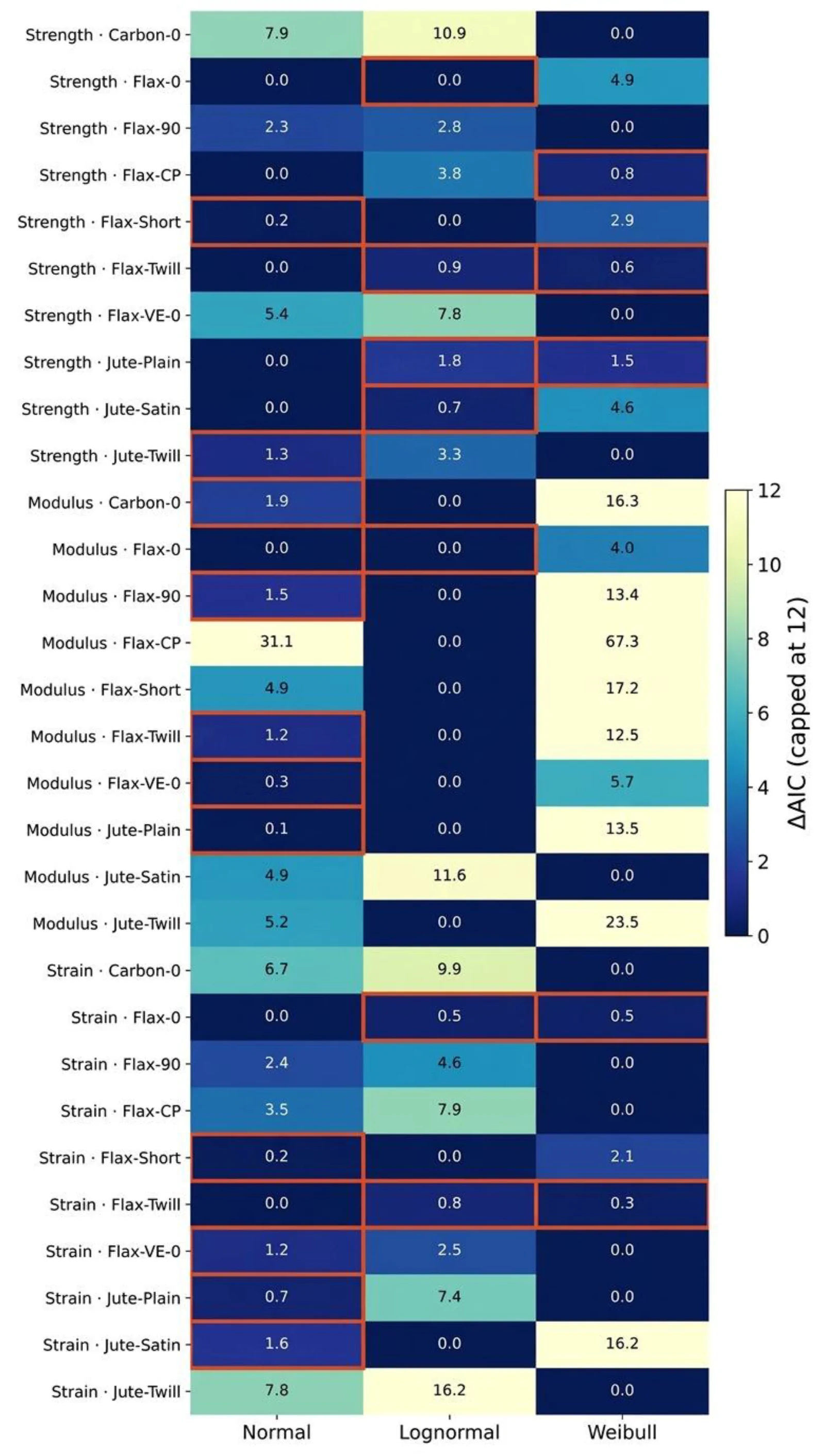
ΔAIC of the normal, lognormal, and Weibull families for the 30 property–group datasets (Jute-twill strain: 84 eligible records). Zero marks the AIC-selected family; cells outlined in red are near-tied (0 < ΔAIC ≤ 2). Values above 12 shared the top color.

Non-normal families were selected in 22 of the 30 datasets, and 19 of 30 had at least one alternative family within ΔAIC ≤ 2 (Table 1, Figure 1). Therefore, family selection carries appreciable uncertainty at these sample sizes. For five datasets, the Anderson–Darling OSL of the AIC-selected family was below 0.05 (Carbon-0 strength, Flax-90 modulus, Flax-CP modulus, Jute-Twill modulus, and Flax-CP strain), indicating that the selected distribution did not adequately describe the observed data according to this test. This finding highlights the limitations of relying on AIC alone for distribution selection. In 21 of 30 datasets, the normal distribution was not rejected by the Anderson–Darling test.

### 4.2. Tensile-Strength Characteristic Values

**Figure 2:**
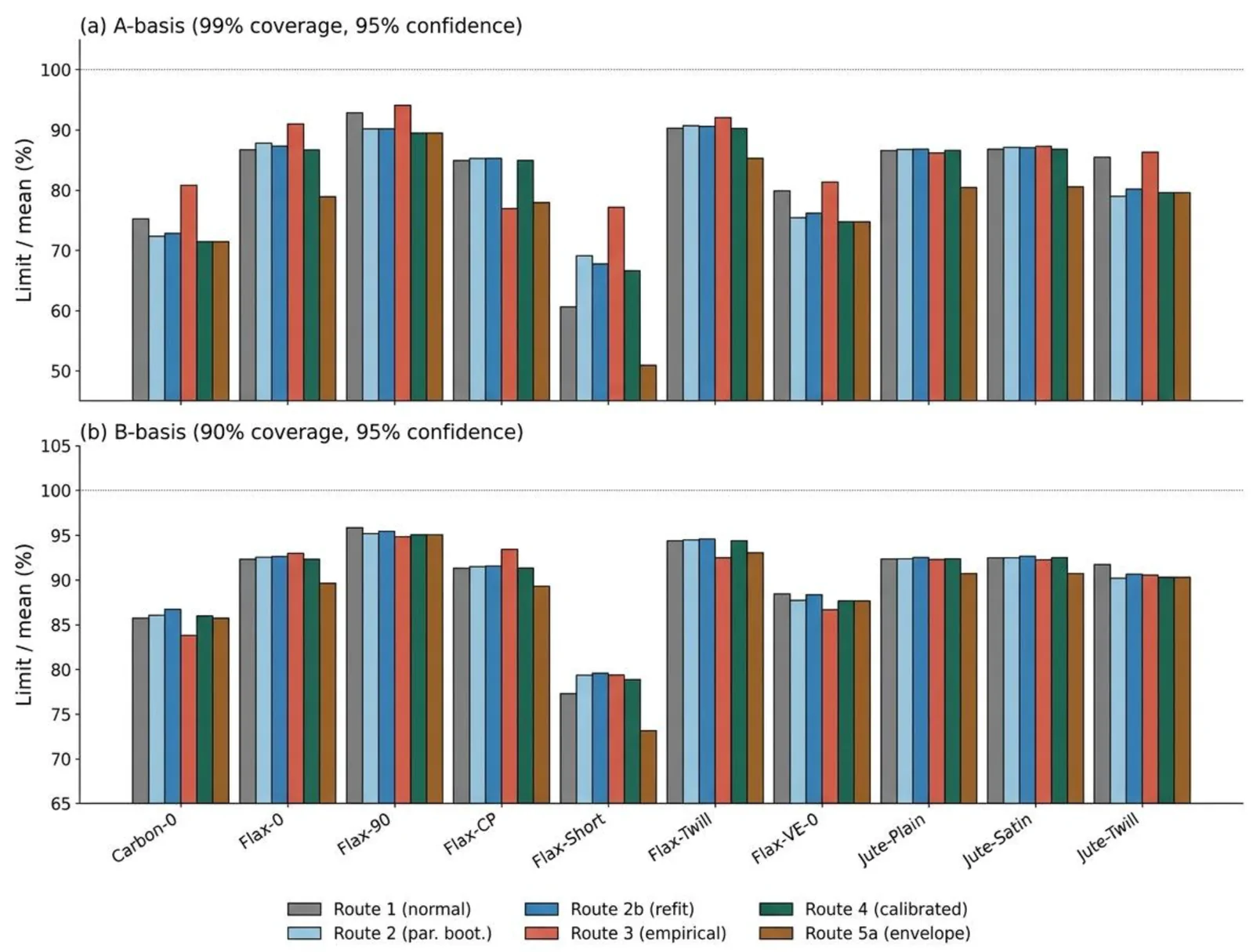
Strength limits expressed as a percentage of the sample mean for (**a**) A-basis and (**b**) B-basis: Route 1 (normal), Route 2 (parametric bootstrap), Route 2b (refitted), Route 3 (empirical), Route 4 (calibrated, AIC-selected family), and Route 5a (minimum over all three families). Group labels are taken from a single list used for all panels.

The A- and B-basis tensile-strength estimates were compared across the statistical routes to assess the influence of distributional assumptions and estimation procedures. Table 2 summarizes the characteristic values for all ten laminate groups, and Figure 2 presents the corresponding limits normalized by the sample mean. The complete route-specific results are provided in Supplementary Table S1.

For the five groups whose strength was normal-selected (Flax-0, Flax-CP, Flax-Twill, Jute-Plain, and Jute-Satin), Route 4 equals Route 1 by construction and agrees closely with Routes 2 and 2b (Table 2, Figure 2). For Weibull- and lognormal-selected groups, Route 4 falls on either side of Route 1. For Carbon-0, the normal-theory A-basis is 885.66 MPa and the Weibull-calibrated value is 841.19 MPa, about 5% lower. For Flax-Short, with the largest strength CV (13.42%) and a lognormal selection nearly tied with normal (ΔAIC = 0.2), the calibrated A-basis is 31.19 MPa versus 28.36 MPa for normal theory. These differences are conditional on the fitted families; they do not show which limit is correct for the experimental population (Section 4.5 and Section 5.2). Route 3 gives the highest A-basis in most groups because it cannot go below the observed minimum (Section 3.2).

### 4.3. Tensile-Modulus Characteristic Values

**Figure 3:**
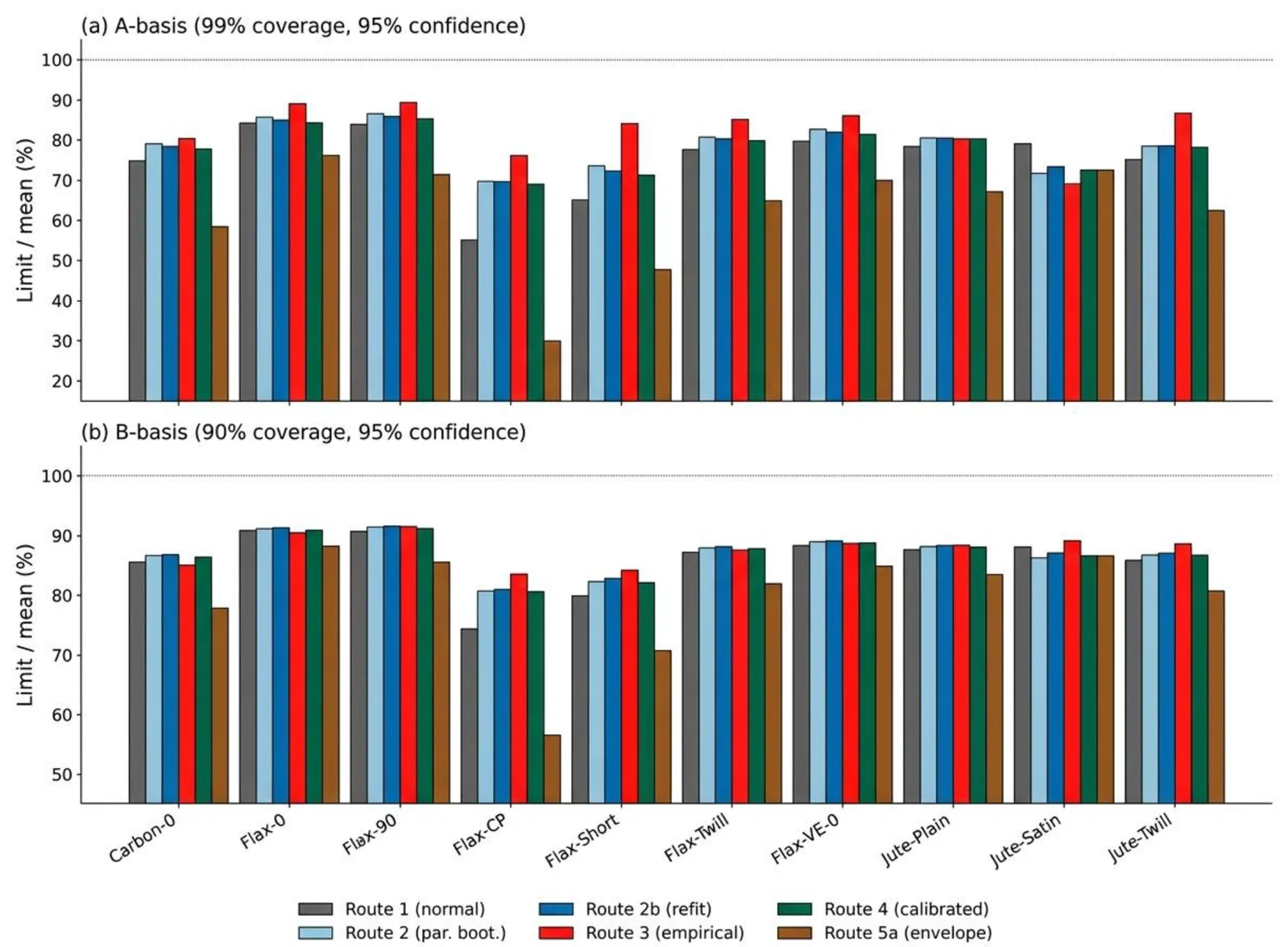
Modulus limits as a percentage of the sample mean: (**a**) A-basis, and (**b**) B-basis; routes as shown in Figure 2.

**Figure 4:**
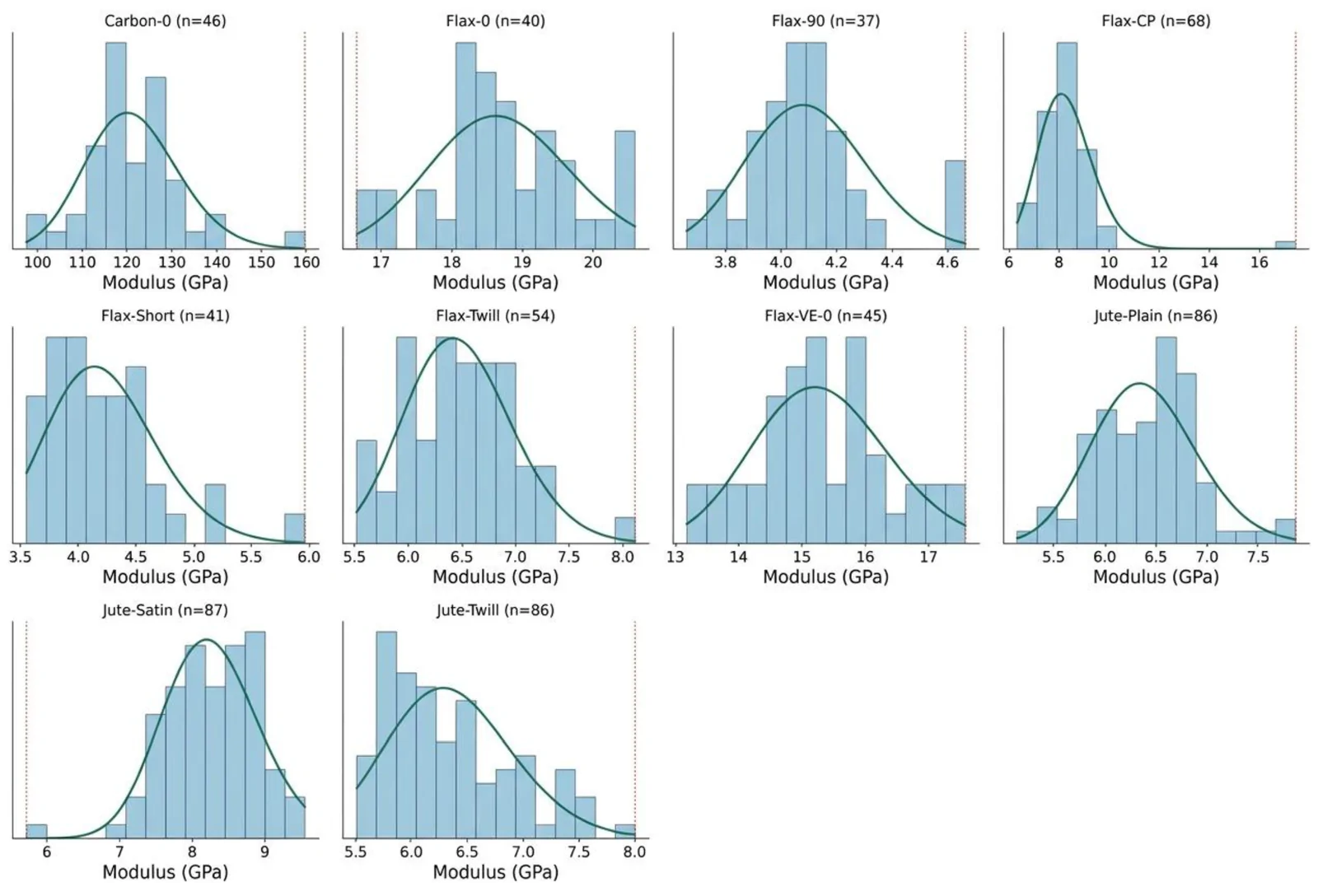
Modulus histograms of the ten groups with fitted lognormal density (green). The dotted red line marks the specimen with the largest absolute standardized residual in each group.

The tensile-modulus characteristic values were evaluated using the same statistical routes as those applied to the strength. Particular attention was given to the influence of distribution selection and individual observations, because the modulus estimates exhibited substantial differences between some fitted models. The calculated limits are presented in Table 3, with normalized comparisons and distributional characteristics shown in Figure 3 and Figure 4, respectively.

Lognormal was selected for eight of the ten modulus datasets (normal for Flax-0 and Weibull for Jute-Satin), but a right-skewed shape is not necessarily an intrinsic property, since modulus is an averaging quantity. Inspection (Figure 4, Supplementary Table S6) shows that the selection is often driven by one or two specimens.

In Flax-CP, the modulus CV is 16.2% and the skewness 4.74; one specimen (Flax-CP_Specimen_RawData_13 (2)) lies 6.8 standard deviations above the mean and is flagged by the MNR test. Removing it reduces the CV to 9.1%, changes the AIC-selected family from lognormal to normal, and moves the calibrated A-basis from 5.745 to 6.119 GPa, while the normal-theory A-basis changes from 4.587 to 6.119 GPa. The gap between normal theory (4.587 GPa) and the calibrated value (5.745 GPa), about 25%, is therefore largely produced by that single specimen, and the Anderson–Darling test rejects the lognormal fit (OSL 0.000). Bimodality coefficients of the ten modulus datasets lie between 0.25 and 0.66, below the conventional 0.555 threshold for all groups except Flax-CP (0.66, driven by the single flagged specimen) with Jute-Twill at the threshold, so we find no strong evidence of bimodality but cannot exclude strain-interval or alignment effects that the dataset does not document.

### 4.4. Failure Strain and the Jute-Twill Records

**Figure 5:**
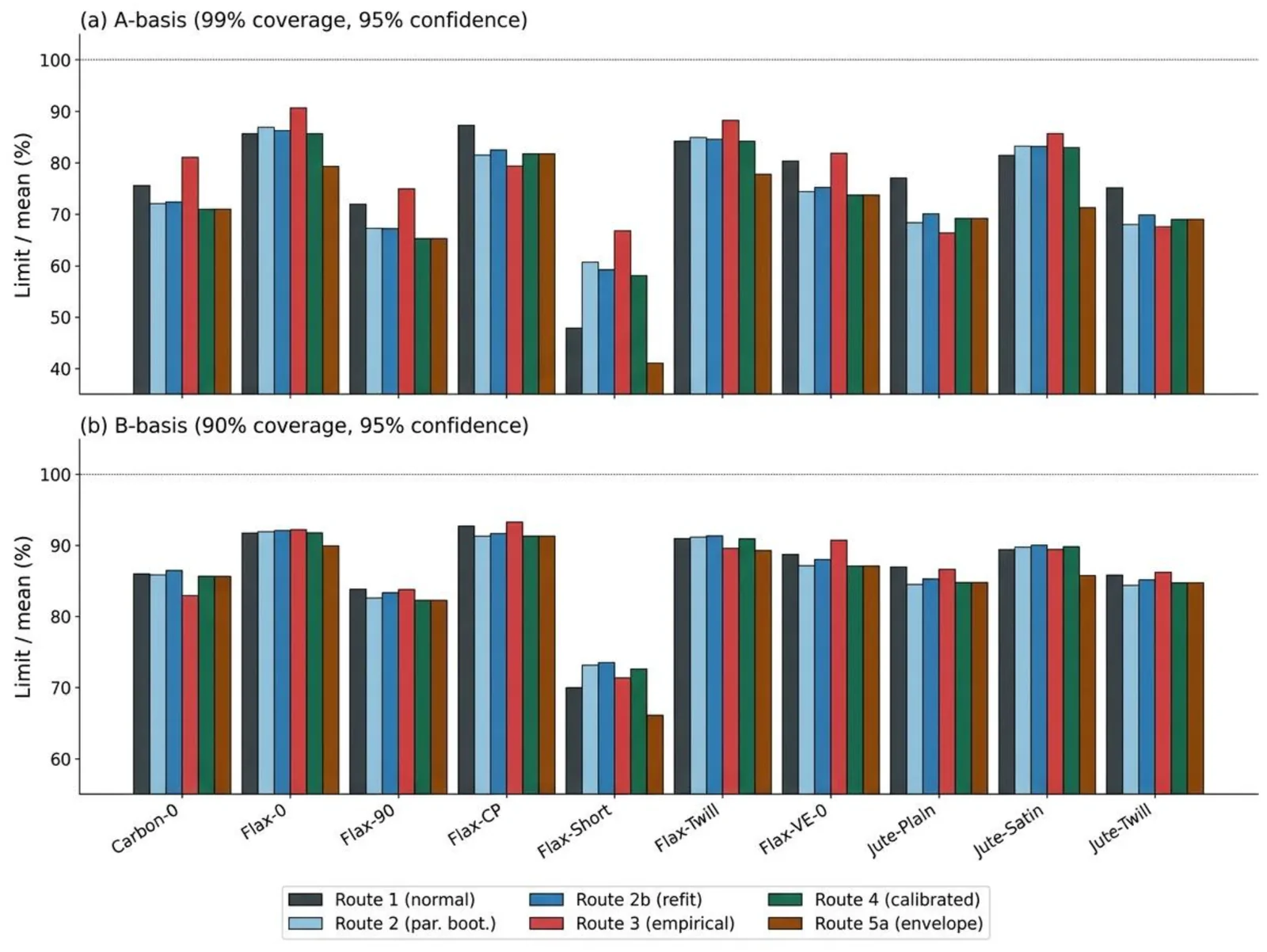
Failure-strain limits as a percentage of the sample mean: (**a**) A-basis, and (**b**) B-basis; Jute-Twill uses the 84 eligible records; routes as in Figure 2.

The failure-strain characteristic values were calculated using video-based strain measurements and the eligibility criterion established in Section 2.2. The primary analysis included 84 eligible Jute-Twill records, whereas the full 86-record dataset and a reconstructed-strain case were considered separately for sensitivity assessment. Table 4 summarizes the characteristic values for the ten laminate groups. The normalized A- and B-basis failure-strain limits for all ten laminate groups are compared in Figure 5.

The Jute-Twill group requires particular attention because two records contained zero video-strain readings at peak stress, resulting in negative processed failure strains. To quantify the influence of these observations, Table 5 compares the original 86-record dataset, the reconstructed-strain sensitivity case, and the primary 84-record dataset.

**Figure 6:**
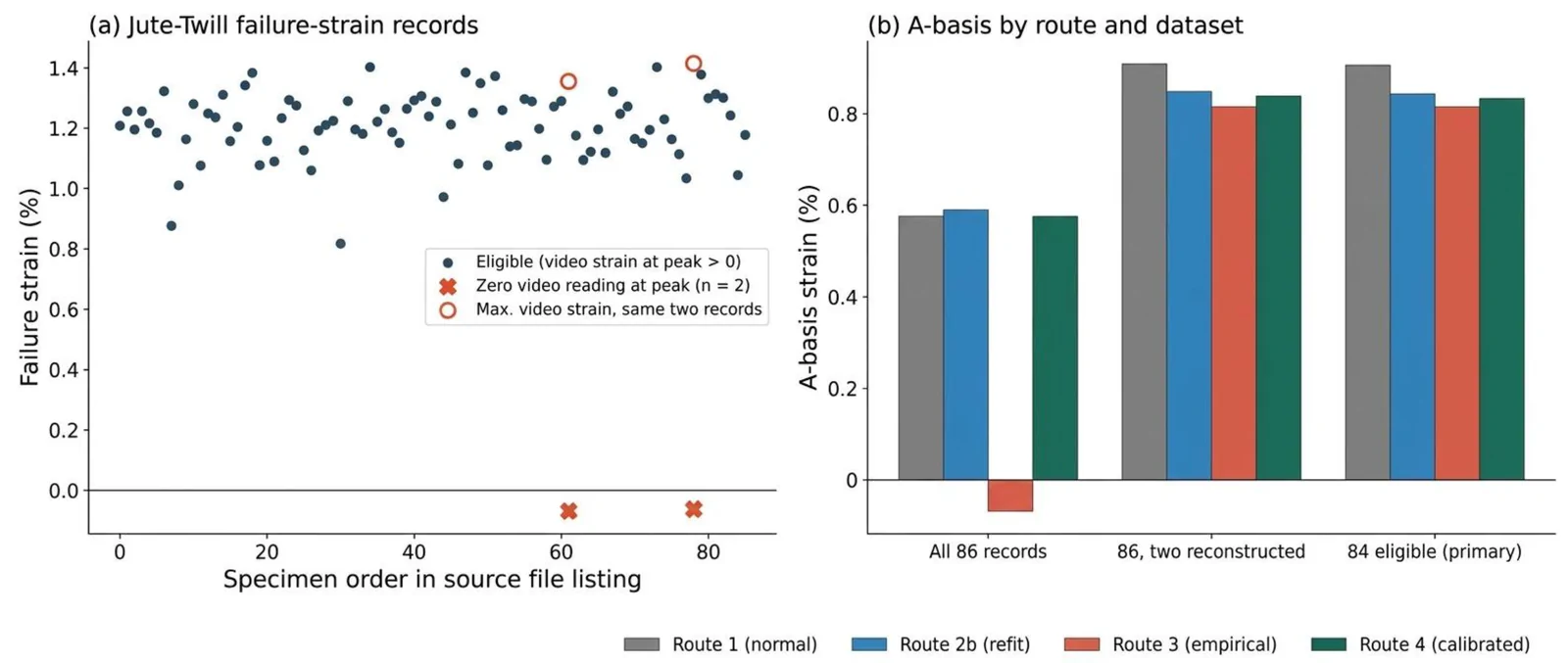
(**a**) Jute-Twill failure-strain records in file-listing order. Crosses: two records with a zero video-strain reading at peak load (processed strain −0.068% and −0.061%); open circles: the maximum video strain recorded during the same tests. (**b**) A-basis failure strain by route for all 86 records, the 86 records with the two strains reconstructed, and the 84 eligible records (primary).

The eligibility rule of Section 2.2 removes two records from the strain analysis (primary *n* = 84). With all 86 records, the CV is 18.8% and normal is the only family that can be fitted to the full sample, because lognormal and Weibull are undefined for negative values; the “selection” is then not a model comparison and Routes 2 and 4 are normal-based by default. With the 84 eligible records, the CV is 9.13%, Weibull is selected (ΔAIC 7.8 versus normal and 16.2 versus lognormal), and the Route-4 A-basis rises from 0.576% to 0.834% (+45%; relative changes computed from unrounded values). The empirical-bootstrap A-basis changes from –0.068% (negative, physically impossible) to 0.816%. The reconstructed sensitivity case gives 0.840%, within 0.6% of the primary result, so the conclusion does not depend on how the two values are treated. A positive Route-4 or Route-1 value on the contaminated sample reflects the mean and standard deviation of 86 values and does not demonstrate robustness; it is simply biased low. The 84-record dataset is used in all primary results, including the family counts, retention, and sample-size analyses; the 86-record sample appears only as a labelled sensitivity case (Table 5, Figure 6).

### 4.5. Sensitivity to Family Selection and to Flagged Specimens

**Figure 7:**
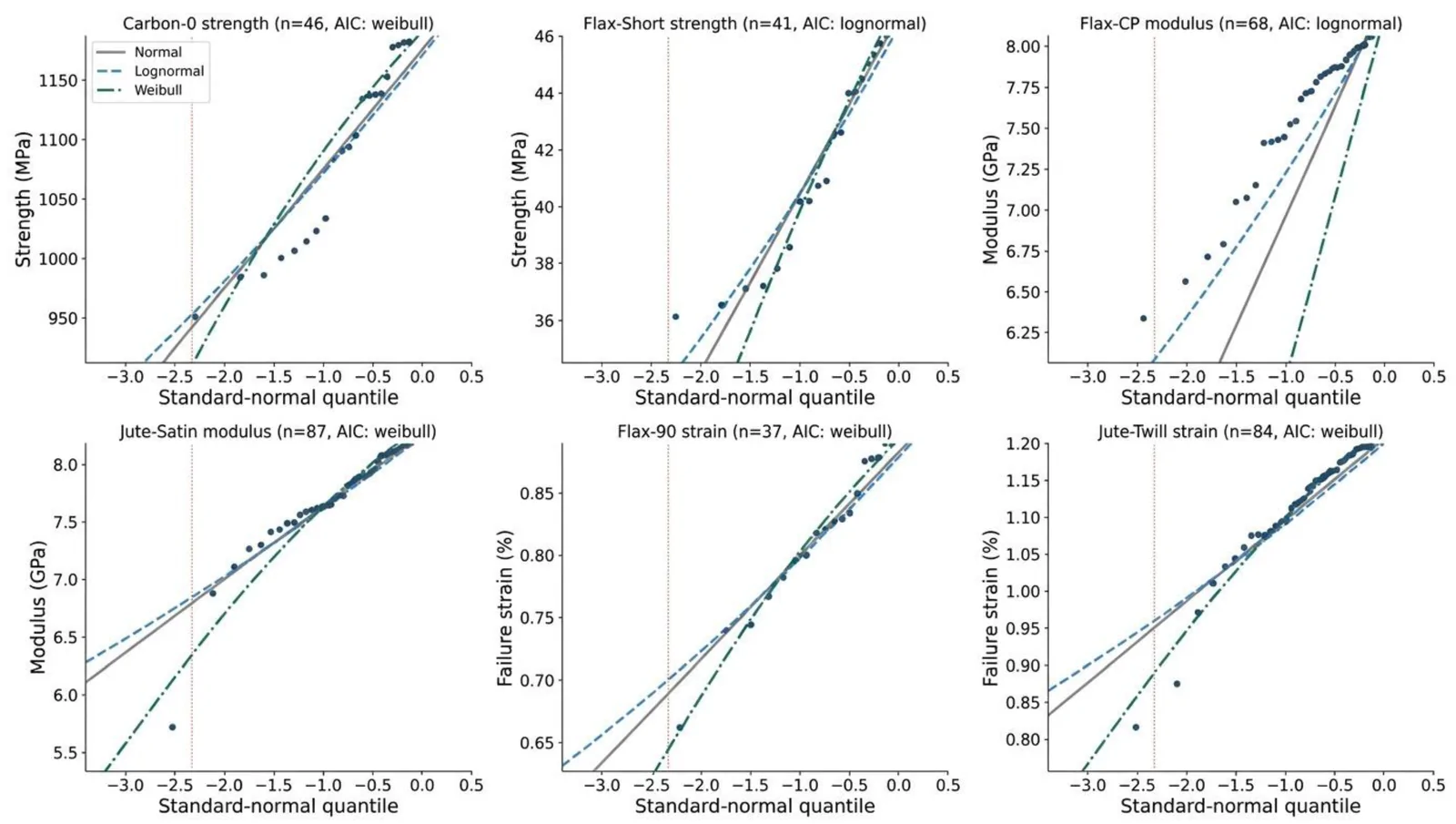
Lower-tail probability plots (Hazen plotting positions) of six datasets with normal, lognormal, and Weibull fits. The dotted line marks the standard-normal quantile of the first percentile. Only the lower part of each distribution is shown.

Because 19 of 30 datasets have a supported alternative, the A-basis was recomputed under each family (Supplementary Table S1). Taking the minimum over the near-tied families (Route 5s) lowers the Route-4 A-basis in 13 of 30 datasets, by up to 17% (median over the 30 datasets 0.0%), and taking the minimum over all three feasible families (Route 5a) lowers it by a median of 7.5% and by as much as 57% (Flax-CP modulus, where the Weibull fit is rejected by ΔAIC = 67 and is not a credible alternative). The engineering consequence of model uncertainty is therefore of the order of a few percent for most datasets and larger for a few, and it is of either sign relative to Route 4. Lower-tail probability plots (Figure 7) show why: in each panel, the lowest observations deviate from at least one fitted line (the two lowest Jute-Twill strains lie below the Weibull line, and, in the Flax-CP modulus, the lowest points depart from all three families), and, with 37 to 87 observations, the first percentile (dotted line) lies at or beyond the smallest observation, so the tail itself is not resolved.

The MNR test flagged at least one specimen in 9 of 30 datasets. Removing the flagged specimens changed the AIC-selected family in 7 of these (Supplementary Table S6), which indicates that single specimens drive the family choice for several datasets, particularly the modulus. Statements such as “normal theory is unnecessarily conservative for Flax-CP modulus” or “optimistic for Carbon-0 strength” are therefore conclusions under the fitted generating models, not findings about the experimental populations.

### 4.6. Coverage Validation

The statistical routes were evaluated through Monte Carlo simulations to determine whether their estimated lower tolerance limits achieved the nominal 95% confidence requirement. Eight generating scenarios covering normal, lognormal, and Weibull populations were considered, with 5000 replicates per scenario. The generating parameters, sample sizes, and population quantiles are listed in Table 6.

**Figure 8:**
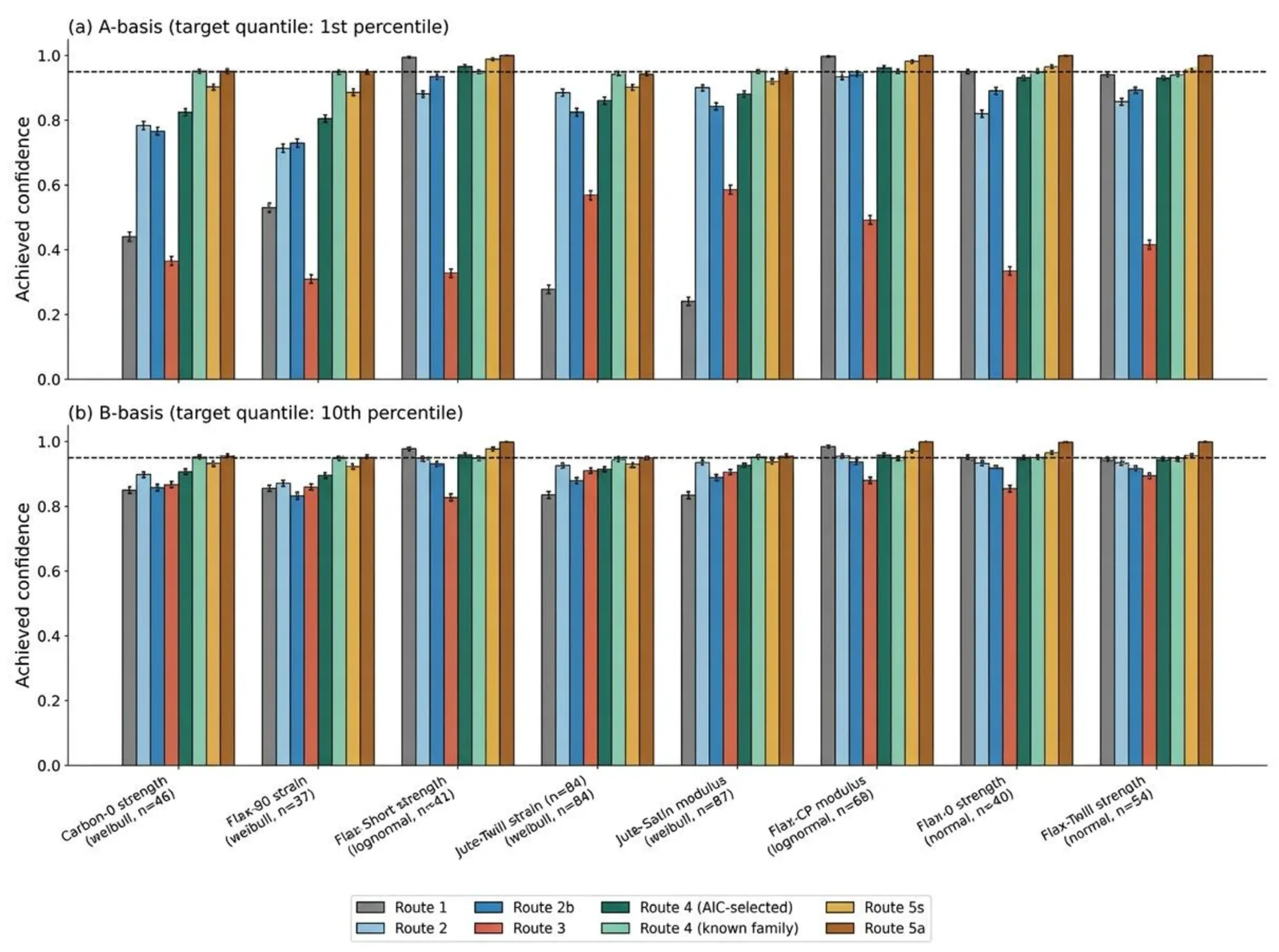
Achieved confidence (fraction of 5000 replicates with the limit at or below the true population quantile) for (**a**) A-basis and (**b**) B-basis in the eight scenarios; error bars are ±1.96 Monte Carlo standard errors. The dashed line is the nominal 0.95. Route 4 (known family) uses the generating family; Routes 2, 2b, 4 (AIC), 5s, and 5a use the family selected by AIC in each replicate.

When the generating family is known, Route 4 achieves nominal confidence: the A-basis confidence lies between 0.943 and 0.952 in all eight scenarios (mean 0.949), within 0.007 (at most 2.3 Monte Carlo standard errors) of 0.95 (Table 7 and Figure 8). When the family must be selected from the data, this property is lost: the mean A-basis confidence of Route 4 is 0.896 and the minimum is 0.805 (Carbon-0 strength 0.825, Flax-90 strain 0.805, Jute-Twill strain 0.861, and Jute-Satin modulus 0.881), and only two of eight scenarios reach 0.94. At a confidence of 0.80, the stated requirement fails in 20% of repeated datasets instead of 5%, and, with a Monte Carlo standard error of 0.003, this cannot be attributed to simulation noise. The Weibull-selected values in Table 2, Table 3 and Table 4 should therefore be read as approximate 80–90% bounds, not as 95% bounds. The simulation does not establish that Route 4 under AIC selection satisfies the nominal requirement; the 95% interpretation is valid only for a family fixed in advance. The same applies to the B-basis (mean 0.932, minimum 0.896). For the two normal and two lognormal scenarios, Route 4 under AIC selection achieved confidence values ranging from 0.932 to 0.967. Although these results were generally closer to the nominal target than those obtained for the Weibull scenarios, values below 0.95 still indicate a failure to satisfy the required confidence. Thus, the relatively small effect of family mis-selection in these scenarios should not be interpreted as evidence of nominal calibration.

The corresponding B-basis results are presented in Table 8. Although the less extreme population percentile generally reduces the severity of the calibration shortfall, family selection continues to affect the achieved confidence. Therefore, the results require separate interpretation for the A- and B-basis targets.

To compare the routes beyond individual scenarios, Table 9 summarizes their mean and minimum achieved confidence, deviation from the nominal target, number of scenarios reaching at least 0.94 confidence, and median ratio of the estimated limit to the true population quantile. These measures distinguish confidence shortfalls from conservatism, which cannot be adequately assessed using mean confidence alone. The number of scenarios with an achieved confidence of at least 0.94 is reported as a descriptive indicator of proximity to the nominal target, allowing for Monte Carlo uncertainty. It is not a substitute for the formal 0.95 confidence requirement, which is assessed separately for each scenario.

The remaining routes showed greater deviations from the nominal confidence target. Route 1 achieved a mean A-basis confidence of 0.672 (minimum 0.242), although its achieved confidence exceeded 0.99 in the lognormal scenarios, indicating conservative limits under those generating assumptions. Route 2 achieved a mean confidence of 0.847, while Route 2b, which refits the distribution within each bootstrap sample, achieved 0.854. Thus, refitting did not eliminate the confidence shortfall, suggesting that the discrepancy cannot be attributed solely to the use of empirical quantiles in Route 2. Route 3 achieved a mean confidence of 0.425, with scenario-specific values of approximately 0.31–0.59. This poor performance is consistent with the structural limitation imposed by the sample minimum, for which the theoretical A-basis confidence ceiling ranges from approximately 0.31 to 0.58 for the sample sizes considered (Section 3.2), rather than being attributable to Monte Carlo noise.

**Figure 9:**
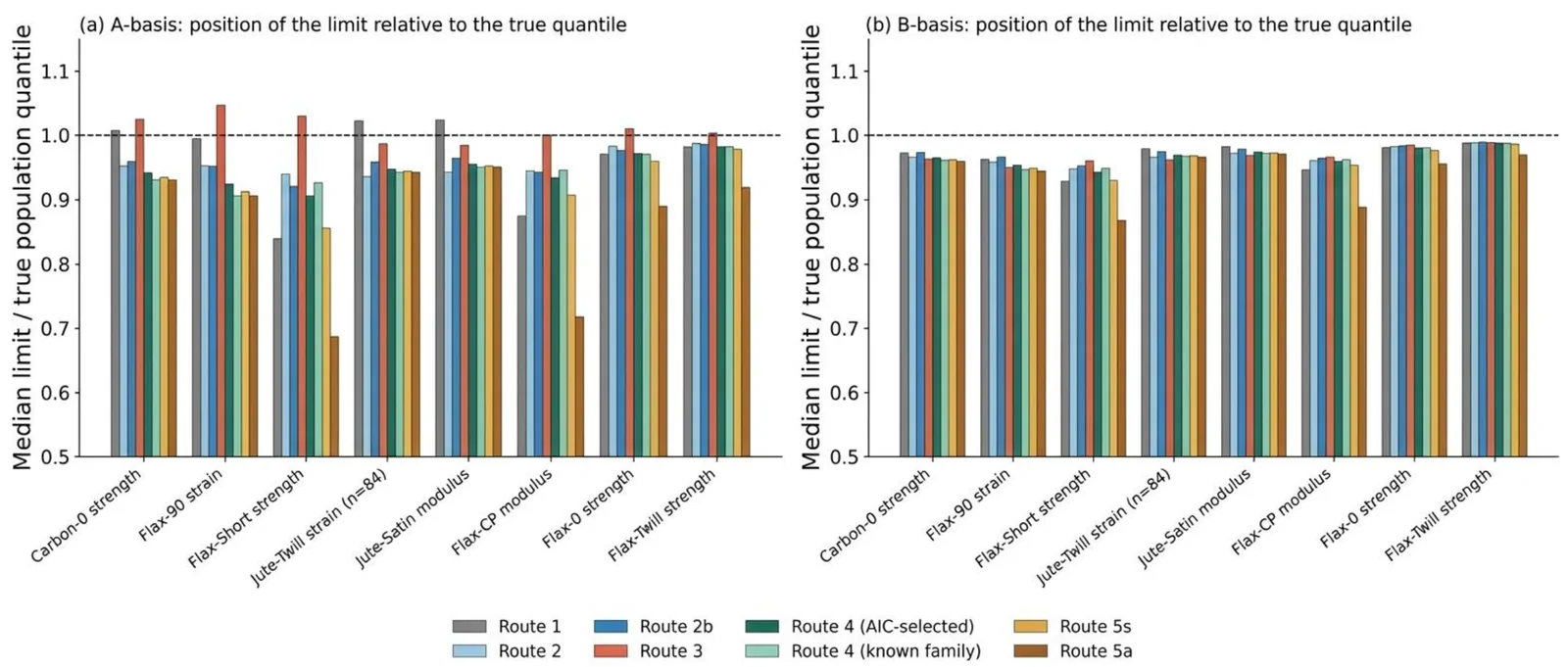
Median ratio of the limit to the true population quantile for (**a**) A-basis, and (**b**) B-basis. Values below one are conservative.

Route 5a achieved a confidence of at least 0.944 across all scenarios (mean 0.975), whereas Route 5s achieved a mean of 0.938 (minimum 0.886). However, the improved confidence of Route 5a was accompanied by greater conservatism in the estimated limits, with a median limit-to-true-quantile ratio of 0.912 compared with 0.944 for Route 4 under the known-family condition (Figure 9). In the normal and lognormal scenarios, Route 5a achieved a confidence close to or equal to 1.000 because it selected the minimum limit across all feasible distribution families. Its minimum achieved a confidence of 0.944 is 0.006 (1.9 Monte Carlo standard errors) below 0.95, a level of agreement similar to Route 4 with the known family (minimum 0.943, 2.3 standard errors); both routes were judged by the same criterion.

**Figure 10:**
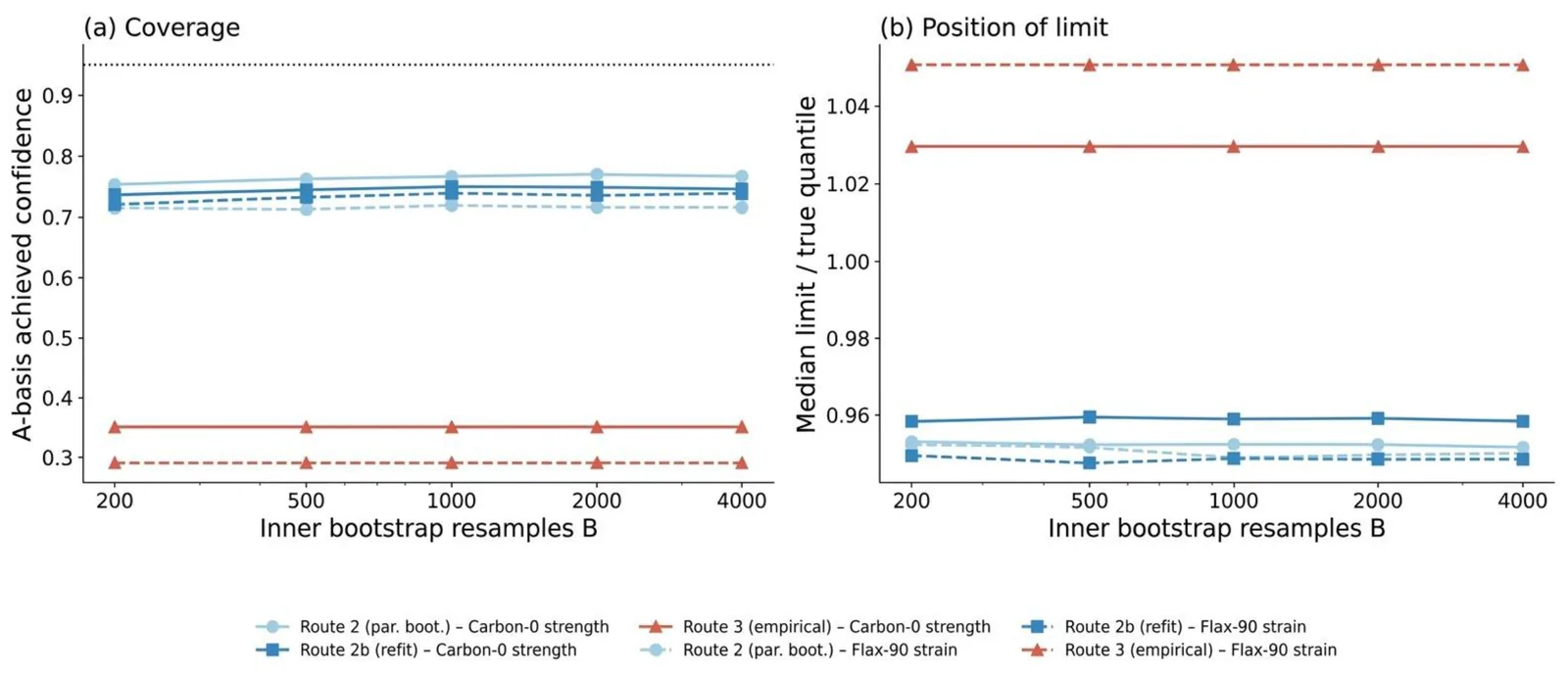
Effect of the number of inner bootstrap resamples B on (**a**) A-basis confidence and (**b**) the median limit/true-quantile ratio for Routes 2, 2b, and 3 in the Carbon-0 strength (solid) and Flax-90 strain (dashed) scenarios (1000 replicates each).

The sensitivity of the bootstrap-based routes to the number of inner resamples was evaluated using two representative scenarios (Figure 10). For Routes 2 and 2b, the achieved A-basis confidence changed by less than 0.01 between B = 500 and B = 4000; for example, Route 2 in the Carbon-0 strength scenario changed from 0.763 to 0.767.

Route 3 remained unchanged because its lower endpoint was determined by the smallest sample observations. These results indicate that increasing the number of bootstrap resamples had little effect on the estimated confidence in the two scenarios examined. However, they do not establish convergence for every material–property combination.

### 4.7. Sample Size, Subsampling and the Empirical-Bootstrap Limit

**Figure 11:**
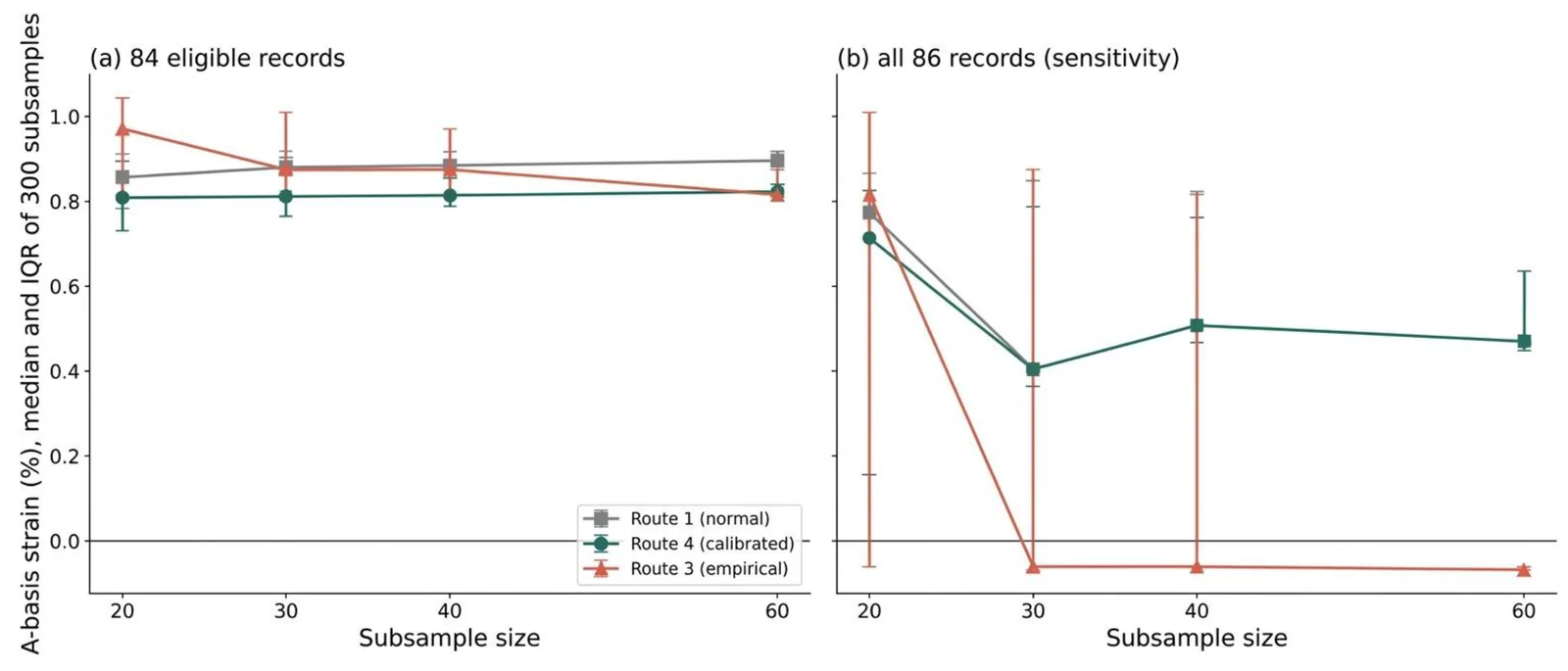
Median and interquartile range of the A-basis failure strain of Jute-Twill over 300 random subsamples without replacement: (**a**) the 84 eligible records, and (**b**) all 86 records. Routes 1, 3, and 4 (family selected within each subsample).

Figure 11 shows the median and interquartile range, over 300 random subsamples drawn without replacement, of the A-basis failure strain of Jute-Twill for subsample sizes 20–60. The statistic plotted is the A-basis of each subsample (Routes 1, 3, and 4 with family selected in the subsample); the bands describe the subsampling variability conditional on this dataset and are not confidence limits on the population tolerance limit. For the 84 eligible records, the median Route-4 value was 0.81% at *n* = 20 and 0.82% at *n* = 60, with no negative Route-3 values. For the contaminated 86-record sample, the median Route-3 value was positive at *n* = 20 (0.82%) and non-positive at *n* = 30, 40, and 60 (−0.06%, −0.06%, and −0.07%); the fraction of subsamples with a non-positive value was 0.39 at *n* = 20 and 0.92 at *n* = 60. The empirical result is therefore not negative for every sample size; it is positive at *n* = 20.

### 4.8. Retention Comparisons and Series Structure

The characteristic value retention was examined to compare the statistical reduction from the sample mean across laminate groups. Because retention depends on both material variability and sample size, the analysis distinguishes estimates based on the actual specimen counts from estimates recalculated at a common sample size of *n* = 40. Table 10 presents the corresponding strength retention values and their rankings.

**Figure 12:**
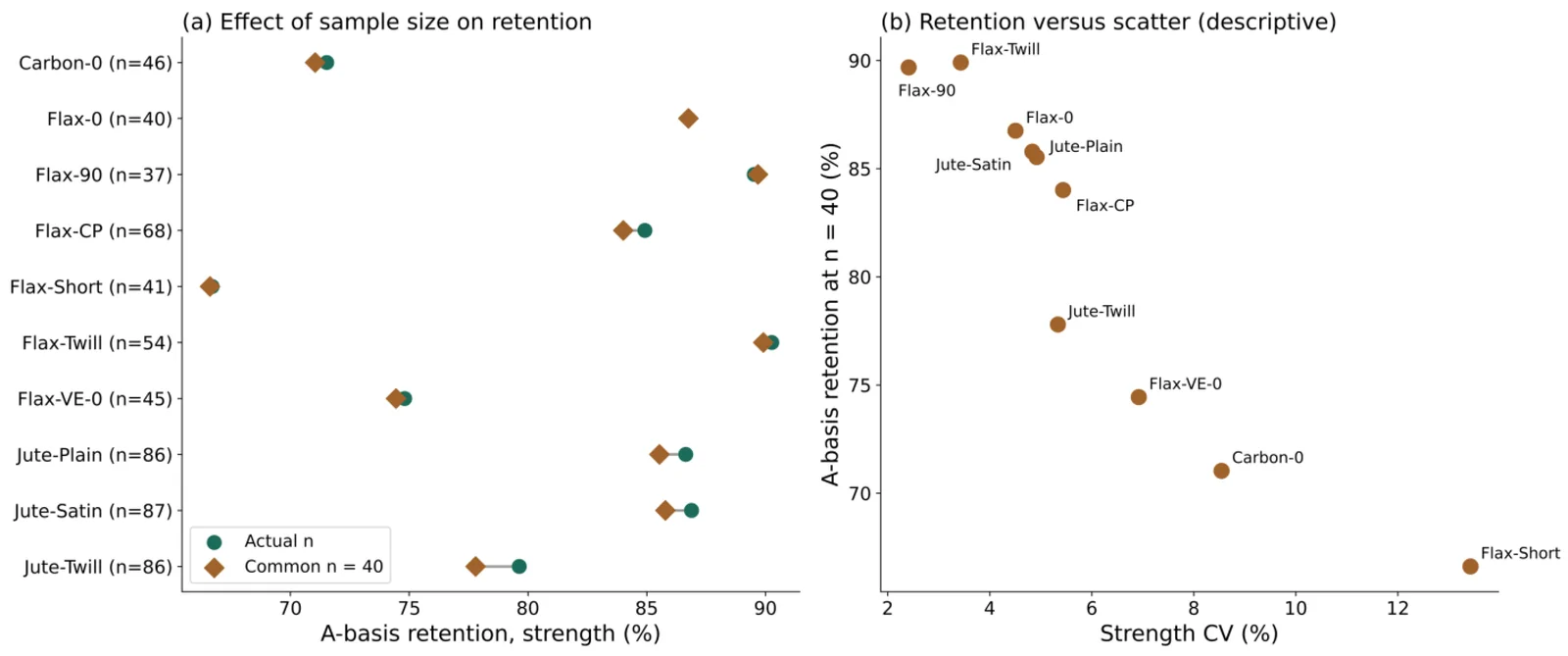
(**a**) A-basis strength retention at the actual sample size and at *n* = 40. (**b**) Retention at *n* = 40 versus strength CV (descriptive analysis).

Retention is a descriptor of the calculated penalty for the present evidence, not an intrinsic material attribute, because the tolerance factor depends on *n*. Calibrated A-basis strength retention ranged from 66.7% (Flax-Short) to 90.3% (Flax-Twill), and B-basis retention from 78.9% to 95.1% (Table 10, Figure 12). Equalizing the sample size to *n* = 40 changes the ordering of the two normal-selected groups: Jute-Satin (CV 4.83%, *n* = 87) ranks above Flax-0 (CV 4.50%, *n* = 40) at the actual sizes (86.9% versus 86.8%) but below it at *n* = 40 (85.8% versus 86.8%). Carbon-0 retained 71.5% despite having the highest mean strength. The negative relationship between retention and CV (Figure 12b) follows partly from the construction and is descriptive only. The ordering at the actual n is a ranking of the current evidence; comparisons of material variability should use the common-*n* values, and downselection should not rest on retention alone.

An exploratory comparison was also conducted between the two filename-defined specimen series, which were distinguished by the presence or absence of the suffix “(2)”. Within the five groups containing this suffix, the tensile strength differed significantly between the two series in every group (Welch *p* between 0.0000 and 0.008; Anderson–Darling k-sample *p* ≤ 0.005). The tensile modulus differed in four of the five groups (*p* < 0.03; Jute-Plain *p* = 0.20), whereas the failure strain differed only in Jute-Satin. These differences suggest that the pooled datasets may contain distinct subpopulations, potentially affecting the estimated standard deviations and characteristic values. However, the filename suffix is an undocumented grouping proxy, rather than a confirmed manufacturing batch identifier. Therefore, this exploratory analysis should not be interpreted as a formal CMH-17 batch variability assessment, which requires documented information on the batch. The resulting characteristic values were interpreted as conditional estimates derived from the pooled single-source specimens. For strength, the normal-theory A-basis computed separately for each series, relative to the pooled value, was +2.5% and −2.5% (Flax-CP), −2.0% and +4.7% (Flax-Twill), −0.6% and −0.3% (Jute-Plain), +4.8% and +0.8% (Jute-Satin), and +2.5% and −3.1% (Jute-Twill) for series 1 and 2, respectively (series 1 = no “(2)” suffix); the direction was not uniform, and the effect on the strength limits was small compared with the family selection effects (Supplementary Table S7).

## 5. Discussion

### 5.1. What the Evidence Supports

This study provides a reproducible assessment of the nominal tolerance-limit confidence when the family must be chosen from the same data. Three results stand on their own. First, the pivotal limits reach the nominal confidence only when the family is fixed in advance (mean 0.949); under AIC selection, the confidence falls to 0.896 on average and to 0.805 in the worst scenario, so the 95% interpretation is conditional on a correctly specified family. Second, a limit that ignores the information shortage in the lower tail cannot be rescued by resampling: the empirical bootstrap is capped at a confidence of 1 − 0.99^*n*^, and the parametric bootstrap benchmarks, with or without refitting, are no better calibrated than the pivotal limit. Third, a conservative envelope over plausible families (Route 5a) brings the confidence to at least 0.944 (within two Monte Carlo standard errors of 0.95) in every scenario but is over-conservative when the family is in fact normal or lognormal. A practitioner with a single-source sample therefore has a choice between a limit that is calibrated if the family is right and one that is robust to the choice at a visible cost. For near-normal groups, all routes agree closely, and the added machinery does not create artificial differences.

### 5.2. Family Selection and the Claims That Can Be Made

AIC identifies the preferred model through overall likelihood and does not validate the experimental lower tail. Nineteen of 30 datasets have a near-tied alternative, the Anderson–Darling test rejects the selected family for five, and flagged specimens change the selected family in seven (Section 4.1 and Section 4.5). Synthetic populations generated from the selected fits are a legitimate way to study operating characteristics under those assumptions, but they provide no independent evidence that the assumptions describe the real material tails. For this reason, the differences between the normal-theory and calibrated limits are reported as consequences of the fitted models. The direction of the normal-theory difference cannot be predicted from first principles: it is negative for the Weibull-selected Carbon-0 strength and positive for the lognormal-selected Flax-CP modulus, but the latter depends on a single specimen. Where the engineering decision matters, Route 5a or a fixed pre-specified family should be reported alongside the AIC-selected value.

### 5.3. Data Eligibility

The two Jute-Twill records with a zero-video reading at the peak-load sample show that a data-quality problem can be revealed by a divergent empirical bootstrap, but the decision to exclude them rests on the measurement record (a zero reading at the peak sample while the video signal is present elsewhere in the test) and on a rule based solely on the measurement record and independent of the tolerance-limit results. That rule removes only the strain values; the strength and modulus remain valid. We recommend computing an empirical-bootstrap diagnostic alongside distribution-based estimates for open datasets of unknown provenance, while recognizing that its negative result alone does not identify the cause.

### 5.4. Material Scatter and Architecture

The retention results are consistent with the broader understanding that natural fiber composite variability is multiscale. Plant fibers vary with growth conditions, extraction, local geometry, microfibrillar structure, defects, and moisture content, whereas laminate-level scattering depends on the orientation, textile architecture, resin infiltration, voids, and interfacial quality [1,18,19]. In the present study, Flax-Short combined the highest strength CV with the lowest retention, and Flax-Twill and Flax-90 combined low CV with high retention. The Weibull strength is often rationalized by the weakest-link failure of a defect population; however, the dataset contains no fiber volume fraction, void content, fractography, or panel-level information; therefore, we do not attribute the selected families to microstructural mechanisms. The associations are dataset-specific and sensitive to thesample size (Section 4.8). Material selection should also consider the absolute characteristic values, stiffness, density, durability, manufacturing constraints, and loading direction.

### 5.5. Limitations and Future Research Directions

The present study provides a systematic framework for estimating and comparing A-/B-basis characteristic-value approximations for natural-fiber composite laminates using experimentally derived tensile data. By integrating distribution screening, multiple tolerance-limit estimation routes, Monte Carlo validation, sensitivity analyses, and sample-size-adjusted comparisons, the study offers useful insights into the influence of statistical assumptions, distribution-selection uncertainty, and specimen variability on the lower-tail mechanical performance. The analysis also demonstrates the importance of measurement-data verification and provides a reproducible basis for preliminary material screening and comparative reliability assessment.

Nevertheless, certain limitations provide opportunities for future research. The dataset originates from a single experimental program without explicit batch, panel, processing, or environmental identifiers, and the observed differences between the file-name series indicate the need for the further investigation of specimen independence and possible production-related variability. Furthermore, the Monte Carlo validation considers normal, lognormal, and Weibull populations generated from fitted parameters, while the actual experimental lower-tail distributions remain uncertain. Although Anderson–Darling goodness-of-fit and maximum-normed-residual procedures were applied, formal CMH-17 batch-level assessments, including k-sample tests and ANOVA, could not be performed because the necessary identifiers were unavailable. Future studies should therefore incorporate independently manufactured batches, detailed processing and environmental information, and broader distributional assessments to evaluate the generalizability and statistical confidence of the estimated limits. Accordingly, the present characteristic values should be regarded as preliminary screening and comparison estimates rather than substitutes for CMH-17-qualified multi-batch design allowables.

## 6. Conclusions

This study applied established one-sided tolerance-limit procedures to a 590-specimen, ten-group natural-fiber composite dataset and evaluated them using Monte Carlo simulation with stored outputs.

(1) With the generating family known, the pivotal limit reached the nominal confidence (mean A-basis 0.949); with the family selected by AIC from the same sample, it reached 0.896 on average and 0.805 at worst; therefore, the 95% interpretation holds only for a pre-specified family.
(2) The empirical bootstrap cannot exceed a confidence of 1 − 0.99^*n*^ (0.311 at *n* = 37, 0.583 at *n* = 87), and a nonparametric 99%/95% bound requires *n* ≥ 299; refitted and non-refitted parametric bootstraps were not better calibrated than the pivotal limit.
(3) A selection-robust envelope over the plausible families reached at least 0.944 in every scenario but was conservative, with a median limit of 0.912 of the true quantiles.
(4) The model selection was uncertain for 19 of the 30 datasets, and single flagged specimens changed the selected family in seven datasets; differences between normal theory and distribution-specific limits were conditional on the fitted models. In the five groups with a second file-name series, strength differed significantly between the series (Welch *p* ≤ 0.008); therefore, the pooled limits assumed identically distributed specimens, and the series-wise normal-theory A-basis strengths of the lower series ranged from −3.1% to +0.8% relative to the pooled values.
(5) Two Jute-Twill records with a zero-video reading at peak load were excluded from the strain analysis by a rule based solely on the measurement record, which raised the A-basis failure strain by 45% (Route 4).
(6) Strength retention (66.7% to 90.3% for the A-basis) is a ranking of the present evidence, and the two normal-selected groups swap order when the sample size is equalized. The characteristic values are suitable for screening and early-stage comparison and are not design allowables.

## Figures and Tables

**Table 1:** Descriptive statistics, AIC-selected family, near-tied alternatives, and Anderson–Darling OSL of the selected family. The jute twill strain used 84 eligible records.

Property	Group	*n*	Mean	SD	CV (%)	Selected (AIC)	Near-tied (ΔAIC ≤ 2)	AD OSL (Selected)
Strength (MPa)	Carbon-0	46	1176.11	100.49	8.54	Weibull	–	0.013
	Flax-0	40	288.58	13.00	4.50	Normal	Lognormal	0.144
	Flax-90	37	31.17	0.75	2.41	Weibull	–	0.820
	Flax-CP	68	152.70	8.30	5.44	Normal	Weibull	0.164
	Flax-Short	41	46.76	6.28	13.42	Lognormal	Normal	0.403
	Flax-Twill	54	95.81	3.29	3.43	Normal	Lognormal, Weibull	0.208
	Flax-VE-0	45	274.20	18.97	6.92	Weibull	–	0.541
	Jute-Plain	86	71.81	3.53	4.92	Normal	Lognormal, Weibull	0.388
	Jute-Satin	87	107.87	5.21	4.83	Normal	Lognormal	0.264
	Jute-Twill	86	68.22	3.64	5.34	Weibull	Normal	0.247
Modulus (GPa)	Carbon-0	46	121.341	10.528	8.68	Lognormal	Normal	0.070
	Flax-0	40	18.700	0.997	5.33	Normal	Lognormal	0.247
	Flax-90	37	4.096	0.221	5.39	Lognormal	Normal	0.025
	Flax-CP	68	8.310	1.342	16.16	Lognormal	–	0.000
	Flax-Short	41	4.218	0.501	11.87	Lognormal	–	0.170
	Flax-Twill	54	6.477	0.508	7.84	Lognormal	Normal	0.590
	Flax-VE-0	45	15.312	1.068	6.98	Lognormal	Normal	0.764
	Jute-Plain	86	6.400	0.508	7.93	Lognormal	Normal	0.277
	Jute-Satin	87	8.271	0.635	7.68	Weibull	–	0.247
	Jute-Twill	86	6.359	0.579	9.11	Lognormal	–	0.002
Strain (%)	Carbon-0	46	0.930	0.078	8.42	Weibull	–	0.404
	Flax-0	40	1.866	0.091	4.87	Normal	Lognormal, Weibull	0.238
	Flax-90	37	0.883	0.083	9.42	Weibull	–	0.763
	Flax-CP	68	2.142	0.098	4.58	Weibull	–	0.005
	Flax-Short	41	1.176	0.209	17.76	Lognormal	Normal	0.777
	Flax-Twill	54	2.415	0.134	5.56	Normal	Lognormal, Weibull	0.400
	Flax-VE-0	45	2.016	0.136	6.76	Weibull	Normal	0.168
	Jute-Plain	86	1.325	0.111	8.41	Weibull	Normal	0.101
	Jute-Satin	87	1.688	0.115	6.83	Lognormal	Normal	0.401
	Jute-Twill	84	1.207	0.110	9.13	Weibull	–	0.748

**Table 2:** Strength characteristic values (MPa). A-basis: Routes 1 (normal), 2 (parametric bootstrap), 2b (refitted parametric bootstrap), 3 (empirical bootstrap), 4 (calibrated), 5s, and 5a (envelopes). B-basis: Routes 1 and 4 (all routes in Supplementary Table S1). B = 4000 bootstrap resamples.

Group	*n*	Family	R1	R2	R2b	R3	R4	R5s	R5a	R1	R4
Carbon-0	46	Weibull	885.66	851.25	856.99	951.22	841.19	841.19	841.19	1008.90	1011.49
Flax-0	40	Normal	250.34	253.50	252.02	262.79	250.34	250.34	227.74	266.51	266.51
Flax-90	37	Weibull	28.93	28.10	28.11	29.34	27.90	27.90	27.90	29.88	29.64
Flax-CP ^†^	68	Normal	129.68	130.23	130.26	117.60	129.68	119.10	119.10	139.53	139.53
Flax-Short	41	Lognormal	28.36	32.34	31.70	36.12	31.19	28.36	23.80	36.15	36.88
Flax-Twill ^†^	54	Normal	86.48	86.92	86.79	88.27	86.48	81.76	81.76	90.45	90.45
Flax-VE-0	45	Weibull	219.22	206.93	208.94	223.13	205.12	205.12	205.12	242.54	240.45
Jute-Plain ^†^	86	Normal	62.22	62.32	62.37	61.89	62.22	57.83	57.83	66.34	66.34
Jute-Satin ^†^	87	Normal	93.72	93.94	93.94	94.15	93.72	93.72	86.93	99.81	99.81
Jute-Twill ^†^	86	Weibull	58.34	53.88	54.73	58.91	54.33	54.33	54.33	62.59	61.62

Columns 4–10: A-basis; columns 11–12: B-basis. † Group contains the “(2)” file-name series; strength differed between series (Section 4.8), so pooled limits assume identically distributed specimens.

**Table 3:** Modulus characteristic values (GPa); columns as in Table 2.

Group	*n*	Family	R1	R2	R2b	R3	R4	R5s	R5a	R1	R4
Carbon-0	46	Lognormal	90.911	96.029	95.200	97.595	94.494	90.911	71.001	103.82	104.91
Flax-0	40	Normal	15.767	16.027	15.892	16.654	15.767	15.767	14.252	17.008	17.008
Flax-90	37	Lognormal	3.440	3.544	3.520	3.662	3.495	3.440	2.930	3.717	3.735
Flax-CP ^†^	68	Lognormal	4.587	5.796	5.797	6.337	5.745	5.745	2.492	6.180	6.701
Flax-Short	41	Lognormal	2.749	3.108	3.053	3.552	3.013	3.013	2.018	3.371	3.464
Flax-Twill ^†^	54	Lognormal	5.036	5.232	5.209	5.523	5.179	5.036	4.210	5.650	5.689
Flax-VE-0	45	Lognormal	12.216	12.663	12.563	13.185	12.483	12.216	10.709	13.529	13.599
Jute-Plain ^†^	86	Lognormal	5.021	5.153	5.159	5.146	5.141	5.021	4.304	5.614	5.641
Jute-Satin ^†^	87	Weibull	6.548	5.940	6.076	5.721	6.011	6.011	6.011	7.289	7.166
Jute-Twill ^†^	86	Lognormal	4.785	4.992	5.001	5.512	4.977	4.977	3.976	5.462	5.520

Columns 4–10: A-basis; columns 11–12: B-basis. † Group contains the “(2)” file-name series; strength differed between series (Section 4.8), so pooled limits assume identically distributed specimens.

**Table 4:** Failure-strain characteristic values (%); columns as in Table 2. Jute-twill uses 84 eligible records; the sensitivity cases are listed in Table 5.

Group	*n*	Family	R1	R2	R2b	R3	R4	R5s	R5a	R1	R4
Carbon-0	46	Weibull	0.703	0.670	0.674	0.754	0.660	0.660	0.660	0.799	0.797
Flax-0	40	Normal	1.599	1.623	1.610	1.692	1.599	1.481	1.481	1.712	1.712
Flax-90	37	Weibull	0.636	0.594	0.594	0.662	0.576	0.576	0.576	0.740	0.726
Flax-CP ^†^	68	Weibull	1.870	1.746	1.769	1.702	1.752	1.752	1.752	1.987	1.956
Flax-Short	41	Lognormal	0.564	0.715	0.697	0.786	0.683	0.564	0.483	0.823	0.854
Flax-Twill ^†^	54	Normal	2.034	2.051	2.044	2.132	2.034	1.879	1.879	2.196	2.196
Flax-VE-0	45	Weibull	1.621	1.500	1.517	1.651	1.488	1.488	1.488	1.788	1.756
Jute-Plain ^†^	86	Weibull	1.022	0.906	0.930	0.880	0.918	0.918	0.918	1.152	1.123
Jute-Satin ^†^	87	Lognormal	1.375	1.406	1.405	1.447	1.401	1.375	1.204	1.509	1.516
Jute-Twill ^†^	84	Weibull	0.907	0.821	0.844	0.816	0.834	0.834	0.834	1.036	1.023

† Group contains the “(2)” file-name series; strength differed between series (Section 4.8); therefore, pooled limits assume identically distributed specimens.

**Table 5:** Jute-twill failure strain: cleaned descriptive statistics and A-basis by route for the contaminated, reconstructed, and eligible datasets.

Dataset	*n*	Mean (%)	SD (%)	CV (%)	Selected	A: R1	A: R2b	A: R3	A: R4
All 86 records	86	1.177	0.221	18.80	Normal	0.576	0.590	−0.068	0.576
86 records, two strains reconstructed	86	1.210	0.110	9.11	Weibull	0.910	0.849	0.816	0.840
84 eligible records (primary)	84	1.207	0.110	9.13	Weibull	0.907	0.844	0.816	0.834

**Table 6:** Simulation scenarios: generating parameters (strength in MPa, modulus in GPa, strain in %), sample size, analytic population quantiles, and fraction of replicates in which the AIC selected the generating family.

Scenario	Generating Family	*n*	Parameters	True A-Basis Quantile	True B-Basis Quantile	Family Selected Correctly
Carbon-0 strength	Weibull	46	β = 15.79, η = 1219	910.9	1057	0.84
Flax-90 strain	Weibull	37	β = 12.97, η = 0.9191	0.6447	0.7727	0.79
Flax-Short strength	Lognormal	41	μ = 3.836, σ = 0.1351	33.85	38.98	0.68
Jute-Twill strain (*n* = 84)	Weibull	84	β = 13.41, η = 1.254	0.89	1.06	0.91
Jute-Satin modulus	Weibull	87	β = 15.46, η = 8.55	6.349	7.392	0.92
Flax-CP modulus	Lognormal	68	μ = 2.108, σ = 0.1297	6.088	6.971	0.74
Flax-0 strength	Normal	40	μ = 288.6, σ = 13	258.3	271.9	0.40
Flax-Twill strength	Normal	54	μ = 95.81, σ = 3.288	88.16	91.59	0.45

The A-basis confidence achieved for each route and scenario is presented in Table 7. The known-family and AIC-selected experiments were distinguished to isolate the effect of distribution selection on calibration. This comparison is particularly important because a method calibrated for a specified family does not necessarily retain nominal confidence when that family is estimated from the same observations.

**Table 7:** Achieved A-basis confidence (target 0.95); 5000 replicates per scenario; Monte Carlo standard error ≤ 0.007. R4 known: generating family; all other routes except R1 and R3 use the AIC-selected family.

Scenario	R1	R2	R2b	R3	R4 Known	R4 AIC	R5s	R5a
Carbon-0 strength	0.441	0.784	0.766	0.366	0.952	0.825	0.903	0.952
Flax-90 strain	0.531	0.714	0.729	0.310	0.949	0.805	0.886	0.949
Flax-Short strength	0.995	0.882	0.936	0.328	0.950	0.967	0.988	1.000
Jute-Twill strain (*n* = 84)	0.279	0.886	0.826	0.569	0.944	0.861	0.903	0.944
Jute-Satin modulus	0.242	0.900	0.843	0.586	0.951	0.881	0.920	0.951
Flax-CP modulus	0.997	0.932	0.945	0.492	0.951	0.963	0.981	1.000
Flax-0 strength	0.951	0.820	0.892	0.335	0.951	0.933	0.965	1.000
Flax-Twill strength	0.943	0.857	0.894	0.416	0.943	0.932	0.956	1.000

**Table 8:** Achieved B-basis confidence (target 0.95); 5000 replicates per scenario; Monte Carlo standard error ≤ 0.007. R4 known: generating family; all other routes, except R1 and R3, use the AIC-selected family.

Scenario	R1	R2	R2b	R3	R4 Known	R4 AIC	R5s	R5a
Carbon-0 strength	0.850	0.898	0.858	0.866	0.952	0.907	0.931	0.956
Flax-90 strain	0.856	0.871	0.833	0.861	0.948	0.896	0.923	0.952
Flax-Short strength	0.978	0.946	0.931	0.828	0.949	0.959	0.977	0.999
Jute-Twill strain (*n* = 84)	0.836	0.927	0.880	0.910	0.945	0.914	0.926	0.949
Jute-Satin modulus	0.835	0.936	0.890	0.906	0.954	0.927	0.939	0.956
Flax-CP modulus	0.985	0.955	0.937	0.880	0.949	0.959	0.971	1.000
Flax-0 strength	0.952	0.933	0.919	0.855	0.952	0.952	0.965	0.998
Flax-Twill strength	0.946	0.933	0.916	0.894	0.946	0.946	0.957	0.999

**Table 9:** Summary of the achieved confidence over the eight scenarios. *L*/*q* is the limit divided by the true population quantile (values < 1 are conservative).

Basis	Route	Mean	Minimum	Mean abs. dev. from 0.95	Scenarios ≥ 0.94	Median *L*/*q*
A-basis	Route 1	0.672	0.242	0.301	4/8	0.988
	Route 2	0.847	0.714	0.103	0/8	0.948
	Route 2b	0.854	0.729	0.096	1/8	0.959
	Route 3	0.425	0.310	0.525	0/8	1.007
	Route 4, known family	0.949	0.943	0.002	8/8	0.944
	Route 4, AIC-selected	0.896	0.805	0.061	2/8	0.944
	Route 5s	0.938	0.886	0.035	4/8	0.939
	Route 5a	0.975	0.944	0.026	8/8	0.912
B-basis	Route 1	0.905	0.835	0.062	4/8	0.975
	Route 2	0.925	0.871	0.026	2/8	0.966
	Route 2b	0.895	0.833	0.055	0/8	0.974
	Route 3	0.875	0.828	0.075	0/8	0.964
	Route 4, known family	0.950	0.945	0.003	8/8	0.965
	Route 4, AIC-selected	0.932	0.896	0.022	4/8	0.967
	Route 5s	0.949	0.923	0.019	4/8	0.965
	Route 5a	0.976	0.949	0.027	8/8	0.957

**Table 10:** Strength retention (limit/mean × 100) at the actual sample size and at a common *n* = 40 with ranks.

Group	*n*	CV (%)	Family	A-Basis Retention (%)	Rank	At *n* = 40 (%)	Rank	Normal Theory, *n* = 40 (%)	B-Basis Retention (%)
Carbon-0	46	8.54	Weibull	71.5	9	71.0	9	74.9	86.0
Flax-0	40	4.50	Normal	86.8	4	86.8	3	86.8	92.4
Flax-90	37	2.41	Weibull	89.5	2	89.7	2	92.9	95.1
Flax-CP ^†^	68	5.44	Normal	84.9	6	84.0	6	84.0	91.4
Flax-Short	41	13.42	Lognormal	66.7	10	66.6	10	60.5	78.9
Flax-Twill ^†^	54	3.43	Normal	90.3	1	89.9	1	89.9	94.4
Flax-VE-0	45	6.92	Weibull	74.8	8	74.4	8	79.7	87.7
Jute-Plain ^†^	86	4.92	Normal	86.6	5	85.5	5	85.5	92.4
Jute-Satin ^†^	87	4.83	Normal	86.9	3	85.8	4	85.8	92.5
Jute-Twill ^†^	86	5.34	Weibull	79.6	7	77.8	7	84.3	90.3

† Group contains the “(2)” file-name series; strength differed between series (Section 4.8), so pooled limits assume identically distributed specimens.

## Data Availability

The raw data supporting this study are publicly available in Mendeley Data, “Statistical behavior of the tensile properties of natural fiber composites,” version 1, DOI: 10.17632/v25pzywt5c.1. The processed specimen-level data, statistical estimates, fitted distribution parameters, calibration factors, Monte Carlo validation results, and sensitivity analyses are presented in Supplementary Tables S1–S7. The Python analysis scripts, unit tests, stored simulation outputs, and raw record verification files are provided in the Code Archive accompanying this submission to facilitate the independent reproduction of the reported analyses. Additional details are available from the corresponding author upon reasonable requests.

## Supplementary Materials

The following supporting information can be downloaded at: https://www.mdpi.com/article/10.3390/polym18212713/s1. The following supporting information accompanies this article: Supplementary Tables S1–S7, containing characteristic-value estimates, specimen-level processed data, fitted distribution parameters, Weibull calibration factors, Monte Carlo validation and convergence results, outlier and subsampling analyses, and exploratory specimen-series comparisons. A separate Code Archive provides the Python analysis scripts, unit tests, stored simulation outputs, and raw-record verification files used in the computational analyses.

## References

[1] Baley C., Gomina M., Breard J., Bourmaud A., Davies P. (2020). Variability of Mechanical Properties of Flax Fibres for Composite Reinforcement. A Review. Ind. Crops Prod..

[2] Torres J.P., Vandi L.-J., Veidt M., Heitzmann M.T. (2017). Statistical Data for the Tensile Properties of Natural Fibre Composites. Data Brief.

[3] Virk A.S., Hall W., Summerscales J. (2010). Failure Strain as the Key Design Criterion for Fracture of Natural Fibre Composites. Compos. Sci. Technol..

[4] Li X., Wang F. (2016). Effect of the Statistical Nature of Fiber Strength on the Predictability of Tensile Properties of Polymer Composites Reinforced with Bamboo Fibers: Comparison of Linear- and Power-Law Weibull Models. Polymers.

[5] (2012). Composite Materials Handbook–17 (CMH-17). Polymer Matrix Composites: Guidelines for Characterization of Structural Materials; CMH-17-1G.

[6] Owen D.B. (1963). Factors for One-Sided Tolerance Limits and for Variables Sampling Plans.

[7] Tiwari S., Lee J., Khan M.F., Park N. (2026). Statistical Variability and Lower-Tail Performance Assessment of Tensile Properties in Flax, Jute, and Carbon Fiber Composite Laminates. Polymers.

[8] Thoman D.R., Bain L.J., Antle C.E. (1970). Maximum Likelihood Estimation, Exact Confidence Intervals for Reliability, and Tolerance Limits in the Weibull Distribution. Technometrics.

[9] Lawless J.F. (2003). Statistical Models and Methods for Lifetime Data.

[10] Hahn G.J., Meeker W.Q. (1991). Statistical Intervals: A Guide for Practitioners.

[11] Makam S., Lee Y.-L., Attibele P. (2013). Estimation of One-Sided Lower Tolerance Limits for a Weibull Distribution Using the Monte Carlo Pivotal Simulation Technique. SAE Int. J. Mater. Manuf..

[12] Torres J.P., Vandi L.-J., Veidt M., Heitzmann M.T. (2017). The Mechanical Properties of Natural Fibre Composite Laminates: A Statistical Study. Compos. Part A Appl. Sci. Manuf..

[13] Efron B., Tibshirani R.J. (1994). An Introduction to the Bootstrap.

[14] Vangel M.G. (1994). One-Sided Nonparametric Tolerance Limits. Commun. Stat. Simul. Comput..

[15] Hanson D.L., Koopmans L.H. (1964). Tolerance Limits for the Class of Distributions with Increasing Hazard Rates. Ann. Math. Stat..

[16] Burnham K.P., Anderson D.R. (2004). Model Selection and Multimodel Inference.

[17] Stephens M.A. (1974). EDF Statistics for Goodness of Fit and Some Comparisons. J. Am. Stat. Assoc..

[18] Yan L., Chouw N., Jayaraman K. (2014). Flax Fibre and Its Composites—A Review. Compos. B Eng..

[19] Virk A.S., Hall W., Summerscales J. (2012). Modulus and Strength Prediction for Natural Fibre Composites. Mater. Sci. Technol..

